# Asymmetric interactions between barley yellow dwarf virus -PAV and wheat dwarf virus in wheat

**DOI:** 10.3389/fpls.2023.1194622

**Published:** 2023-07-11

**Authors:** Thomas Armand, Marlène Souquet, Luâna Korn, Kevin Gauthier, Emmanuel Jacquot

**Affiliations:** PHIM Plant Health Institute Montpellier, University of Montpellier, National Research Institute for Agriculture, Food and the Environment (INRAE), French Agricultural Research Centre for International Development (CIRAD), Institut Agro, French National Research Institute for Sustainable Development (IRD), Montpellier, France

**Keywords:** epidemiology, co-infection, WDV, BYDV-PAV, inoculation success, accumulation, insect mediated transmission

## Abstract

The deciphering of the epidemiology of a plant virus has long been focused on the study of interactions between partners of one pathosystem. However, plants are exposed to numerous viruses which lead to frequent co-infection scenarios. This can change characteristics of virus-vector-host interactions and could impact the epidemiology of viral diseases. Barley yellow dwarf virus-PAV (BYDV-PAV; species: *Luteovirus pavhordei*; genus *Luteovirus*), wheat dwarf virus (WDV; genus *Mastrevirus*) and their respective vectors (BYDV-PAV: e.g. *Rhopalosiphum padi* and WDV: *Psammotettix alienus*) are commonly found in cereal fields. Wheat plants co-infected with BYDV-PAV and WDV have been reported from field surveys, although epidemiological outcomes of BYDV-PAV – WDV interactions *in planta* have not yet been studied. Experiments were carried out to evaluate and compare, through different competition scenarios (i.e. single- and co- (simultaneous and sequential) inoculations), the efficiency of BYDV-PAV and WDV to infect, to accumulate in and to be spread between wheat plants. Moreover, the impact of competition scenarios on the biological parameters of these two viruses was evaluated at different stages of the infection and with plants at different ages at inoculation. Results showed i) that these viruses achieve their infection cycle and their plant-to-plant transmission with different efficiencies and ii) BYDV-PAV – WDV interactions lead to different phenotypes ranging from antagonism to synergism. Finally, when these two viruses share a host, the nature and strength of virus-virus interactions varied depending on the order of virus arrival, stages of the infection cycle and plant age at inoculation. Precisely, the introduction (i.e. co- and sequential inoculation) and infection process (i.e. virus accumulation) of BYDV-PAV in a wheat benefit from the presence of WDV. For the latter, the sympatry with BYDV-PAV exerts opposite pressure on parameters involved in virus introduction (i.e. benefit during sequential inoculation) and spread (i.e. lower transmission efficiency and virus accumulation in co-infected plants). In the context of increased potential exposure of crops to insect vectors, this study participates in a better understanding of the impact of BYDV-PAV and WDV co-infections on biological and ecological parameters of the diseases induced by these viruses.

## Introduction

As obligatory parasites, viruses require the host cell machinery to accomplish their infection cycle. Consequently, viruses are not able to replicate and to produce progenies outside permissive cells of compatible hosts. This strict dependency on living organisms represents a challenge for crop viruses for which cultivated hosts are absent during weeks/months following plant removal at harvest. For the maintenance of these parasites in the environment several strategies exist, including abilities i) to be vertically transmitted through the accumulation in seeds, tubers and vegetative organs of host plants (Zinga et al., 2013; Cieniewicz et al., 2017), and ii) to infect several host species, in both cultivated or non-cultivated areas, that have overlapping growing periods over the year (Alexander et al., 2014). Horizontal transmission of viruses that infect rooted hosts could rely on direct contacts (i.e. mechanical inoculations) between neighboring plants and/or on the involvement of vectors (e.g. nematodes, fungi and insects; (Brault et al., 2010)). The biology of these organisms and the nature of their interactions with viruses, allow transmission from short (few meters) to long (up to kilometers) (e.g. transmission of *Plum pox virus* by aphids; (Pleydell et al., 2018)) distances for few minutes/hours (e.g. aphid-borne viruses transmitted in a non-persistent manner; (Brault et al., 2010)) to years (e.g. long-term maintenance of viruliferous status of nematodes; (Demangeat et al., 2005)).

Host infection leads to the production of viral proteins which participate in all steps of the infectious process, including replication, short- and long-distance movement *in planta* and hijacking of both plant defences and metabolism (Alcaide et al., 2022). The efficiency of each of these steps, which depends on the ability of the virus to use the resources of the infected plant, determines the intensity of systemic infection which constitutes the fitness *in planta* of the pathogen (Elena et al., 2014; Alcaide et al., 2020). For plant viruses, fitness varies depending on biotic (host (species, genotype, age…) or pathogen (viral species and isolate, multiple infections…)) and abiotic (linked to the environment (e.g. water, temperature…)) parameters. The wide and intensive network of virus dynamics in the environment exposes host plants to many viral species, strains and isolates. Moreover, host ranges of plant viruses frequently include several species (Moury et al., 2017). This leads to the frequent occurrence of viral co-infections (e.g. Zinga et al., 2013). In co-infections, resources of infected plant are used by each pathogen partner (Elena et al., 2014). Consequently, the fitness *in planta* of a viral entity that infects a host can be i) unchanged (i.e. neutral interaction), ii) increased (i.e. synergistic interaction) or iii) decreased (i.e. antagonistic interaction) by the presence of a co-infecting virus. In return, the fitness *in planta* of the latter is impacted (i.e. symmetric interaction) or not (i.e. asymmetric interaction) by the competitor virus (Alcaide et al., 2020). In addition to raw changes in viral fitness *in planta*, plant physiology can be differentially impacted by single/co-infections (Alcaide et al., 2022) leading to the expression of more or less severe symptoms (Moreno and López-Moya, 2020). For insect-borne viruses, these changes in plant phenotype and physiology may impact performances and/or behaviour of the vectors (Domingo-Calap et al., 2020; Jia et al., 2022). Changes of virus fitness (qualitative and/or quantitative variations of infection rate and/or virus accumulation) and/or vector traits may result in modifications of the frequency and/or the efficiency of transmission events (Péréfarres et al., 2014). Thus, by impacting a wide range of plant-virus-vector interactions, co-infections can alter the infectious process, the severity and/or the spread of viral diseases.

Wheat (*Triticum aestivum*) is one of the four major staple foods cultivated worldwide. This plant species accounts in average for approximately 20% of the calorie intakes in the worldwide human diet (Shiferaw et al., 2013). Therefore, for numerous countries wheat production represents alimentary, economic and geopolitical interests, suggesting that wheat yield should at least be maintained at the current level to match food security. However, wheat is exposed to abiotic (e.g. drought, nutrient deficiency, UV…) and biotic (e.g. bacteria, fungi, viruses, insects…) stresses that can impact grain production. Among viral diseases affecting wheat, yellow dwarf disease (YDD) and wheat dwarf disease (WDD) can induce yield losses of up to 80 and 90%, respectively (Perry et al., 2000; Lindblad and Waern, 2002). YDD can be caused by ten virus, among which the barley yellow dwarf virus-PAV (BYDV-PAV; species: *Luteovirus pavhordei*; genus *Luteovirus*; family *Tombusviridae* (Scheets et al., 2020)) is described to be the most prevalent in France (Lister and Ranieri, 1995). Wheat dwarf virus (WDV; family *Geminiviridae*, genus *Mastrevirus* (Abt and Jacquot, 2015)) is the etiological agent of WDD. These viruses are transmitted in a persistent, non-propagative manner (Brault et al., 2010) by different insect vectors. BYDV-PAV is exclusively transmitted by aphids (mainly *Rhopalosiphum padi* (Linnaeus, 1758) and *Sitobion avenae* (Fabricius, 1775); (Parry, 2013)), whereas, WDDV is transmitted by leafhoppers (*Psammotettix alienus;* (Abt and Jacquot, 2015)). For the last three decades, these viral diseases were not considered an important threat for cereal growers. Indeed, to limit the incidence of YDD and WDD in cereals fields, insects (i.e. aphids and, at a lesser extent, leafhoppers) were efficiently managed in cereal fields with insecticides (Mc Namara et al., 2020). However, the recent ban of neonicotinoids in the EU (Jactel et al., 2019) associated with the description of *Sitobion avenae* clones resistant to pyrethroids in European countries (Fontaine et al., 2023) had a considerable impact on the availability of chemical resources to control insect-borne viral diseases in cereal fields. Thus, the current management methods against YDD and WDD are based on cultural practices (e.g. late sowing, management of volunteers (Östman et al., 2001; Lindblad and Waern, 2002)), the use of limited virus-resistance/tolerance resources (Pfrieme et al., 2022; Pichon et al., 2022) and/or the use of natural-based substances (Pichon et al., 2022). Despite these alternatives, the absence of neonicotinoids-coated seeds (that represented in 2016 in France 35% and 75% of sown wheat and barley seeds, respectively) could increase the exposure of cereals fields to insect-borne viral diseases (Mc Namara et al., 2020).

Wheat plants co-infected by BYDV-PAV and WDV have already been reported from field surveys (Jarošová et al., 2018; Liu et al., 2020). While main biological parameters involved in YDD and in WDD epidemiology have been studied, particularly for YDD (Van den Eynde et al., 2020), little is known about the outcome of BYDV-PAV – WDV interactions in small grain cereals. Based on our knowledge, Jarošová et al. (2018) provided a first attempt to study BYDV-PAV – WDV interactions by monitoring virus load in co-infected plants. Monitoring viral accumulation in infected plants (i.e. a proxy of virus fitness *in planta*) is an important step to study interactions between viruses. However, this approach does not allow *per se* a comprehensive understanding of the impact of co-infections on epidemiology of the disease induced by each viral partner. The aim of our study was to estimate the impact of co-infections on biological parameters involved in the epidemiology of BYDV-PAV and WDV. For each virus, monitored parameters (i.e. infection rate, virus accumulation and transmission efficiency) were evaluated at different steps of infection using single and competitive (i.e. simultaneous and sequential) inoculations.

## Materials and methods

### Plants, vectors and viruses

Wheat plants cv. Rubisko [RAGT, Rodez, France] were used in this study. Seeds were sown in tubes (2cm diameter, 10 cm height; 1 seed/tube) or in pots (Length x width x height: 7x7x7 cm; approximately 30 seeds/pot) containing N2 soil (Neuhaus^®^ Huminsubstrat N2, Klasmann Deilmann, Geeste, Germany) to produce plantlets used in the experiments or in insect rearing systems, respectively. Plantlets were maintained in insect-proof containment at 24°C/20°C (day/night: 16h/8h) for seven days after sowing, before being used in the experiments.

Separated colonies of *R. padi* (clone RpIA, collected in the department of Yonne (France) in 2012) and populations of *P. alienus* (collected in the department of Côte-d’Or (France) in 2012, Abt et al., 2020) were reared in a growth chamber (day/night: 16h/8h, 24°C/20°C, RH: 40%) in the presence of wheat plants.

Isolate 4 of the barley yellow dwarf virus (i.e. BYDV-PAV4 in Chain et al., 2007) and isolate w1 of the wheat dwarf virus (WDV-w1, Abt et al., 2020) were maintained on wheat plants in plexiglass cages in the presence of RpIA and *P. alienus* vectors, respectively. The plants in rearing systems were tested for virus infection by DAS-ELISA before the experimentations. This information was used to validate the characteristics (either reared on healthy, on BYDV-infected, on WDV-infected or on mixed (BYDV and WDV) infected plants) of insects used in the experiments (Abt et al., 2020; Pichon et al., 2022).

### BYDV-PAV/WDV inoculations

#### Single BYDV-PAV and WDV inoculations

Inoculations of wheat plantlets with one virus (BYDV-PAV or WDV) were carried out using 2 insects (sampled from infected rearing systems) per plant (i.e. BYDV-PAV: *R. padi* N_2_-N_3_ nymphs; WDV: *P. alienus* (larvae or adults)) and an inoculation access period (IAP) of 24h. During IAP, each plant was individually maintained under micro-perforated cellophane bag. Control, mock inoculations were carried out with virus-free aphids and leafhoppers. Single inoculations were carried out using wheat plantlets at 7, 9, 12, 15 and 19 days after sowing. At the end of IAP, insects were removed manually and plants were sprayed with insecticide (Pirimor G; 0.1% (v/v); Syngenta, Basel, Switzerland). Plants were then maintained in an insect-free growth chamber (day/night: 16h/8h, 24°C/20°C, RH: 40%) for 21 days before being tested for their sanitary status using serological diagnosis (DAS-ELISA, detailed in a dedicated paragraph). Experiments were carried out with series of 20 plants for each age of plantlets/virus combination and the whole experimental design was repeated 3 times.

#### Simultaneous inoculation

Wheat plantlets (at 7-, 9-, 12-, 15- and 19-days after sowing) were inoculated simultaneously by BYDV-PAV and WDV using *R. padi* (BYDV-PAV: 2 aphids (from BYDV-infected rearing systems) per plant) and *P. alienus* (WDV: 2 leafhoppers (from WDV-infected rearing systems) per plant) and a 24h IAP. During IAP, plants were individually maintained under micro-perforated cellophane bag. At the end of IAP, insects were removed manually. Plants were sprayed with insecticide (Pirimor G; 0.1% (v/v); Syngenta, Basel, Switzerland) and maintained in an insect-free growth chamber for 21 days before being tested for their sanitary status using serological diagnosis (DAS-ELISA, detailed in a dedicated paragraph). Experiments were carried out with series of 20 plants for each set of plants (7-, 9-, 12-, 15- and 19-days old) and the whole experimental design was repeated 3 times.

#### Sequential inoculation

Inoculations of wheat plantlets (at 2-leaf stage, i.e. 7 days after sowing) with one virus (BYDV-PAV or WDV) were carried according to the procedure described for single virus inoculations. Control, mock inoculations were carried out with virus-free aphids and leafhoppers. At the end of IAP, insects were removed manually, and plants were placed in an insect-free growth chamber for 2-, 5-, 8- and 12-days before being used for a second inoculation step. Thus, depending on the insects (aphids for BYDV-PAV or leafhoppers for WDV) used in the first inoculation step, plants were exposed to aphid/BYDV-PAV (for plants previously exposed to leafhopper-mediated inoculation) or to leafhopper/WDV (for plants previously exposed to aphid-mediated inoculation), using the previously described inoculation procedure (i.e. 2 insects/plant; 24h IAP, see above). Based on the type of inoculation (mock inoculation (_M_), BYDV-PAV (_B_) and WDV (_W_)), plants from sequential inoculations were labelled _M/B_, _M/W_, _B/W_ or _W/B_ depending on _first/second_ inoculation steps. At the end of the second IAP, insects were removed manually and plants were sprayed with insecticide (Pirimor G; 0.1% (v/v); Syngenta, Basel, Switzerland). Then, plants were maintained in an insect-free growth chamber for 21 days before being tested for their sanitary status using serological (DAS-ELISA, detailed in a dedicated paragraph) and molecular (RT-qPCR or qPCR detailed in a dedicated paragraph) diagnostic tools. Experiment was carried out with series of 20 plants for each virus/date after the first inoculation (2-, 5-, 8- and 12-days) combination and the whole experimental design was repeated 3 times.

### Virus transmission from BYDV-PAV/WDV singly-and co-infected plants

Insects collected from infected rearing systems (i.e. 5 *R. padi* N_1_-N_4_ nymphs per plant for BYDV-PAV and/or 5 P*. alienus* (adult or larvae) per plant for WDV) were maintained on wheat plantlets (7-days old) under micro-perforated cellophane bag for a 24h IAP. After IAP, insects were carefully removed manually from inoculated plants. These insect-free plants were then transferred in an insect-proof growing chamber. These plants were used as *source* plants in transmission experiments carried out at either 7 or 21 days after the inoculation (DAI) of *source* plants. Virus-free insects (10 *R. padi* for BYDV-PAV or 20 P*. alienus* for WDV) were deposited on each *source* plant for 6 h (BYDV-PAV) and 24 h (WDV) acquisition access periods (AAPs). After the AAPs, insects were transferred on 7-days old healthy wheat *test* plants (1 aphid/*test* plant for BYDV-PAV or 2 leafhoppers/*test* plant for WDV) for 1-week IAP. Insects were then removed manually from *test* plants and plants were sprayed with insecticide (Pirimor G; 0.1% (v/v); Syngenta, Basel, Switzerland). *Source* and *test* plants were maintained in an insect-free growth chamber for 3 weeks after inoculation before being tested for their sanitary status using serological and molecular diagnostic tools. BYDV-PAV transmissions were carried out using 10 BYDV-PAV-infected and 10 co-infected *source* plants for each DAI. WDV transmissions were carried out using 8 WDV-infected and 10 co-infected *source* plants for each DAI. The whole experimental design was repeated 3 times.

### Calculation of rates and ratios

Following virus inoculation, plants can i) remain healthy or ii) become infected. The proportion of infected plants among the inoculated ones corresponds to the infection rate of the virus in single inoculation procedures (*I_Si_*). The total infection rate of each virus (*I_Tot_*) was considered from plants simultaneously inoculated with two viruses. The expected occurrence of co-infections was evaluated by calculating the theoretical co-infection rate (*I_Th_*), i.e. the multiplication of *I_Si_* values of inoculated viruses (Lacroix et al., 2014). The observed co-infection rate (*I_Sim_*) was calculated from simultaneous co-inoculations. Co-infections from sequential inoculations were used to calculate infection rate from pre-infected plants (*I_Seq_*). It is important to note that among sequentially inoculated plants, only plants infected by the first inoculated virus were considered to calculate *I_Seq_* of the competitor virus. Finally, transmission rate of a virus from singly- (*T_Si_*) and co-infected (*T_Ci_*) plants were estimated. Variables listed above are described in **Table 1**. The above listed parameters were used to build total infection (*R_tot_* =*I_Tot/_I_Si_*), co-infection (*R_ci_* =*I_Sim/_I_Th_*), competition (*R_com_* =*I_Seq/_I_Si_*) and transmission (*R_tr_* =*T_Ci/_T_Si_*) ratios (**Table 2**). Ratio values above 1 suggest that involved viruses interact synergistically. Ratios equal or below 1 indicate neutral and antagonistic interaction patterns, respectively.

**Table 1:** Variables used in the study.

Variable	Definition
*I_Si_=* Number of infected plants/ Number of singly inoculated plants	Infection rate after single inoculation
*I_Tot_=* (Number of singly infected plants + Number of co-infected plants) / Number of simultaneously inoculated plants	Total infection rate of a virus
*I_Th_= I_Si_(BYDV-PAV)* x *I_Si_(WDV)*	Theorical co-infection rate according to Lacroix et al.(2014)
*I_Sim_=* Number of co-infected plants/ Number of co-inoculated plants	Co-infection rate after simultaneous inoculation
*I_Seq_=* Number of co-infected plants/ Number of pre-infected plants submitted to second inoculation step	Co-infection rate after sequential inoculation
*T_Si_=* Number of infected plants/ Number of plants inoculated from singly-infected source plant	Transmission rate from singly-infected plants
*T_Ci_=* Number of infected plants/ Number of plants inoculated from co-infected source plant	Transmission rate from co-infected plants

**Table 2:** Variables used in the study.

Variable	Definition
*R_tot_= I_Tot_*/ *I_Si_*	Ratio of total infection
*R_ci_= I_Sim_*/ *I_Th_*	Ratio of co-infection
*R_com_= I_Seq_*/ Infection rate from single inoculation of mock inoculated plants	Ratio of competition
*R_tr_=T_Si_*/ *T_Ci_*	Ratio of transmission

### Kinetics of virus accumulation

Single and simultaneous inoculations of BYDV-PAV and WDV were carried out on 2 leaf-stage wheat plants using insects collected from infected rearing systems (i.e. 5 *R. padi* N_1_-N_4_ nymphs per plant for BYDV-PAV and/or 5 P*. alienus* (adult or larvae) per plant for wdv) and a 24h IAP. At the end of IAP, insects were removed manually and plants were sprayed with insecticide (Pirimor G; 0.1% (v/v)). At different (i.e. at 2, 5, 8 and 12 days) DAI wheat plants were sampled for molecular (BYDV-PAV: RT-qPCR; WDV: qPCR) diagnosis. Inoculations were carried out on series of 60 plants/inoculation procedure (i.e. single and simultaneous inoculations of BYDV-PAV and WDV) to allow sampling series of 15 plants per virus/inoculation procedure/DAI combination. The whole experimental design was repeated 3 times.

### Sampling plants and nucleic acids extraction

The first five centimeters of the apex of each leaf were sampled from each plant to be tested. Sampled material was stored at -20°C before nucleic acid extraction. Frozen samples were grinded at 1500 rpm for 3 min using the 1600 Mini G™ tissues homogenizer (SPEX™ sample prep, Metuchen, USA) in the presence of 350 µL of grinding buffer (RA1 buffer with 1% of β-mercaptoethanol) from *NucleoSpin RNA/DNA Buffer Set* (Macherey-Nagel^©^, Dueren, Germany) and using the *NucleoSpin^®^ RNA plant kit* (Macherey-Nagel^©^, Dueren, Germany) according to manufacturer’s instructions. Total nucleic acids were eluted in 100µl (WDV: total DNA) and 50µl (BYDV-PAV: total RNA) of elute buffer and RNase-free water, respectively, and stored at -20°C until used in molecular diagnostic assays.

### Serological detection (DAS-ELISA)

Aerial parts (leaves except the 5cm from apex previously collected for nucleic acid extraction) of each wheat plant were individually ground with a Pölhane press (MEKU, Villingen-Schwenningen Germany). The presence of BYDV-PAV and WDV in plant sap was tested by double antibody sandwich enzyme linked immunosorbent assay (DAS-ELISA; Clark and Adams, 1977). Briefly, 100µl of carbonate buffer (15 mM Na_2_CO_3_, 35 mM NaHCO_3_, pH = 9.6) supplemented with WDV (1:200 (v:v); DSMZ, Braunschweig, Germany) or BYDV (1:1000 (v:v); PAV52, H. Lapierre, INRAE) antibodies, were deposited in each well of microtitration plates (Thermo Fisher Scientific, Waltham, USA). Then, plates were maintained at 37°C for either 3h (BYDV-PAV) or 4 h (WDV) before being washed three times with PBST buffer (PBS buffer (137 mM NaCl, 8 mM Na_2_HPO_4_, 12H_2_O, 2.7 mM KCl, 1.5 mM KH_2_PO_4_, pH = 7.4) with 0.05% (v/v) Tween 20). This washing procedure was repeated between each step of the DAS-ELISA protocol. Plant sap (100 µL) was added in coated wells and plates were placed at 4°C overnight. In each well, 100µl of conjugate buffer (PBST buffer, 2% (w/v) polyvinylpyrrolidone 40T, 2% (w/v) ovalbumin) with diluted alkaline phosphatase coupled antibodies (@WDV: 1/200 (v/v) or @BYDV: 1/1000 (v/v)) were deposited and incubated at 37°C for either 3h (BYDV-PAV) or 4h (WDV). After a last washing step, wells were filled with 100 µL of p-nitrophenyl phosphate (1 mg/mL) in substrate buffer (1 N di-ethanolamine, pH = 9.8) and placed in the dark at room temperature. After 2h incubation, a micro-plate reader (Multiskan FC, Thermo Fisher Scientific, Waltham, USA) was used to measure the optical density of each reaction at 450nm (OD_450nm_). Detection thresholds were fixed at twice the mean OD_450nm_ value of healthy control plant samples with a minimal threshold of OD_450nm_ = 0.1.

### DNA standards for molecular assays

The plasmid pPAV6 containing the T7 RNApol promotor sequence upstream of the full length genome of BYDV-PAV-IL isolate (Fabre et al., 2003b), was linearized at the 3’end of the viral sequence using the restriction enzyme *SmaI*. The linearized plasmid was purified by phenol/chloroform/isoamyl according to Fabre et al. (2003b) and used for *in-vitro* transcription of the BYDV-PAV genome with the *T7 RiboMAX™ kit* (Promega^©^, Madison, USA) according to manufacturer’s instructions. RNA transcripts were purified with phenol/chloroform/isoamyl precipitation and quantified using UV densitometry (NanoDrop™ 2000, Thermo Fisher Scientific, Waltham, USA). A fraction (2µL) of viral RNA containing 6.4x10^10^ copies/µl was reverse transcribed into cDNA for 1 h at 37°C in the presence of 5 μl RNase-free water, 5 μl of M-MLV 5X Reaction Buffer, 1.4 μl of dNTP, 2 μl of RNasin (Promega^©^, Madison, USA), 1 μl of M-MLV reverse transcriptase (Promega^©^, Madison, USA) and 2 µl BYDV-PAV specific reverse primer (5’-^3119^GCCCAGCGCTTTCAGAC^3135^-3’). The produced cDNA was diluted to obtain a fraction containing 10^8^ copies/µl of the BYDV-PAV sequence. The pBL-WDV-[Enk1] plasmid, a pBluescript (Stratagene, San Diego, USA) derived plasmid containing the full-length genome of WDV-[Enk1] isolate (Kvarnheden et al., 2002), was quantified using UV densitometry and diluted in DNAse/RNase-free water to produce a fraction containing 10^8^ copies/µl of the WDV genome. Ten-fold serial dilutions were used to produce fractions containing from 10^8^ to 10^3^ copies/µL of BYDV-PAV and WDV sequence. These fractions were used in qPCR reaction as standards for the quantification of virus in plant samples.

### Quantitative polymerase chain reactions

Real-time quantitative polymerase chain reactions (qPCR) were carried out in a final volume of 12.5µl containing 6.25µl of LightCycler^®^ 480 Probes Master (Roche, Basel, Switzerland), 1µl (300nM) of virus specific reverse primer, 1µl (300nM) of virus specific forward primer, 1µl (100nM) of virus specific TaqMan^®^ probe and 1µl of a fraction containing targeted nucleic acid (BYDV-PAV and/or WDV). The primers (UnivWDVfw: 5’-^1381^CGCGCTAGGACAGTCACT^1401^-3’ and UnivWDVrv: 5’-^1533^AAGATTGGCTCAAGGATATGACTCC^1509^-3’) and the probe (WDV probe: 5’-6FAM-^1426^AGGCGAACGAGTAGTTGA^1443^-NFQ-MGB) designed by (Gadiou et al., 2012) were used to detect WDV sequences. The detection of BYDV-PAV was carried out using primer pair (fp: 5’-^3070^AAAGCCAACTCt/cTCCGGG^3087^-3’ and rp: 5’-^3119^GCCCAGCGCTTTCAGAC^3135^-3’) and probe

(TMp: 5’-6FAM-^3093^CAAATTCGGCCCCAGTCTATCGCA^3116^-TAMRA) from Fabre et al. (2003b). The qPCR cycle (10 min at 95°C followed by 40 cycles of 15 sec at 95°C and 1 min at 60°C) was run on LightCycler 480^®^ (Roche, Basel, Switzerland). During qPCR, the fluorescence intensity of the reporter dye FAM (494 nm-521 nm) was recorded in each sample. Collected data allowed the calculation of the net increase of fluorescence from the baseline (i.e. normalised reporter: ΔR_n_), which is proportional to the amount of the amplified target. Cycle threshold values (Ct_v_) were calculated with second derivative methods (Guescini et al., 2008) using LightCycler^®^ 480 Software (1.5 version; Roche, Basel, Switzerland). The Ct_v_ were compared to DNA standard to obtain the amount of target sequence in 1µl of the processed sample (*n*). Then, the number of target sequence per mg of wheat leaves (*N*) was calculated following equation 1 where *V* is the total volume of the sample and *m* is the weight of plant leaves.


(1) $$N=\frac{n*V}{m}$$

### Statistical analyses

Statistical approaches were run using R software (4.1.3 version, R foundation, Indianapolis, US). For all the statistical tests significance levels were fixed below 0.05. The effect of the experimental repetition and its interaction with the biological factors (e.g. temporal conditions, virus species or type of infection) on binary data (i.e. *I_Si_*, *I_Tot_ I_Sim_*, *I_Seq_*, *T_Si_* and *T_Ci_*; **Table 1**) were analysed using generalised linear models (GLM) of the binomial family (link function= logit) (formula: binary data ~ biological factor * experimental repetition) and will be presented in case of significant effect. Similar binomial GLM analysis (link function= logit) were carried out to assess the effect of temporal condition (i.e. “plant age” or “days of infection” or “days after mock inoculation” or “length of pre-infection”; formula: binary variable ~ temporal condition), infection associated factors (i.e. virus species (for experiment including exclusively singly or infection type; formula: binary variable ~ infection associated factors) and/or their interactions (formula: binary variable ~ temporal condition*infection associated factors) on binary data. The construction of each GLM is available in **Supplementary Table 1**. Then, estimated marginal mean test (EMM; with the correction of Tukey) was used to assess the significance of the difference between the studied modalities. In addition, for each plant age the *I_Si_* were used to calculate the *I_Th_* (according to Lacroix et al., 2014) which were compared to the corresponding *I_Sim_* with a Chi-squared² test of conformity.

Virus loads were Log_10_-transformed, before testing the normal distribution and heteroscedasticity of the classes by residues analysis. Then, variation of normal-distributed data with infections associated factors the experimental repetition and its interaction with the biological factors (e.g. temporal conditions, virus species or type of infection) were analysed with ANOVA and will be presented in case of significant effect. Variance analysis assessing the effect of infection associated factors (i.e. virus species (for experiment including exclusively singly or infection type; formula: binary variable ~ infection associated factors), temporal conditions (i.e. “days of infection”; (formula: binary variable ~ temporal condition) and/or their interactions (formula: binary variable ~ temporal condition*infection associated factors) were analysed with ANOVA (the construction of each test is available in **Supplementary Table 1**), followed by EMM as *post-hoc* test.

## Results

### Infection, accumulation and transmission of BYDV-PAV and WDV

#### Impact of plant age at inoculation

**Figure 1:**
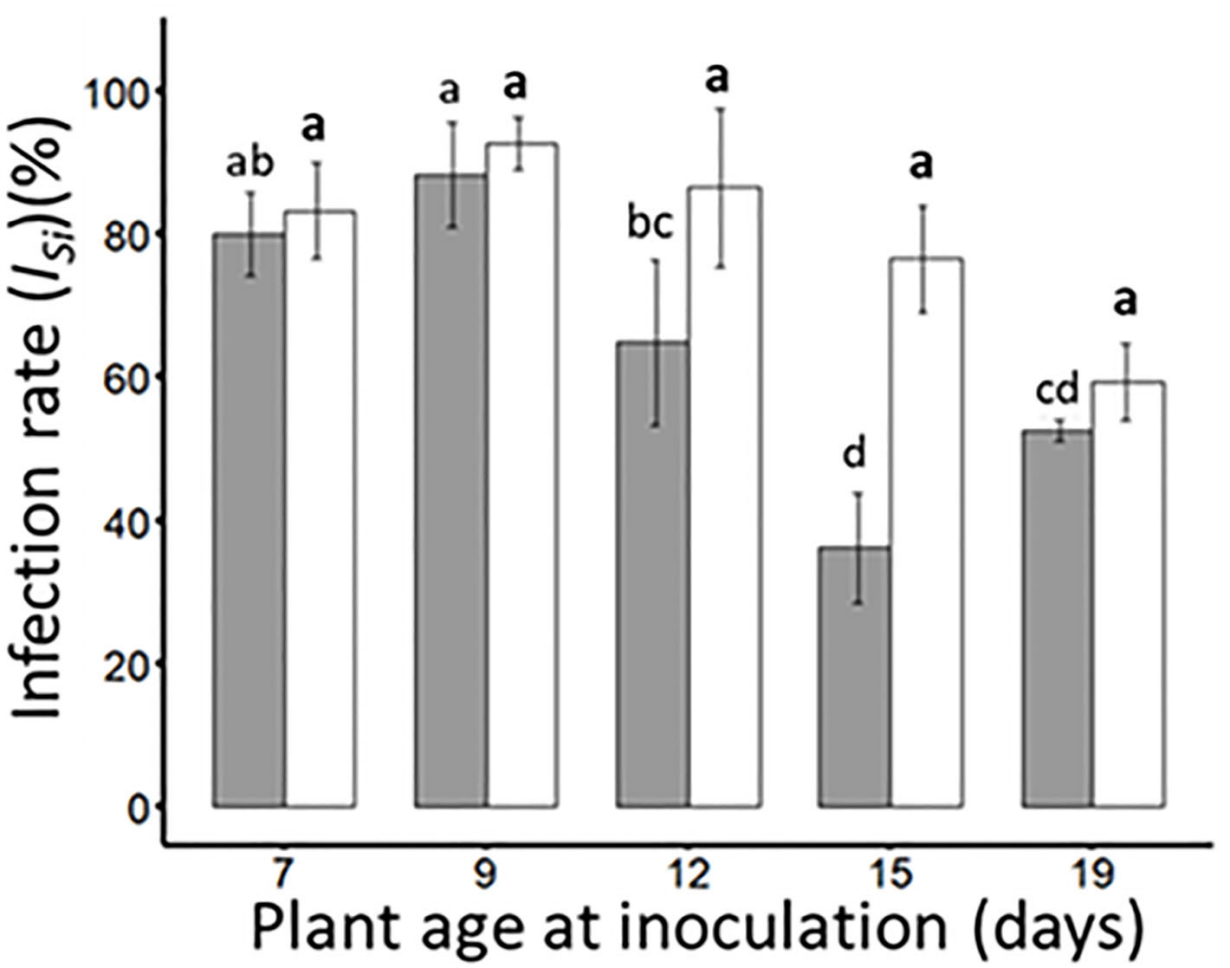
Infection rates of BYDV-PAV and WDV (single inoculation (*I_Si_*)) on wheat plants of different ages at inoculation. Wheat plants of 7-, 9-,12-, 15- and 19-days old were inoculated with either BYDV-PAV (2 *R. padi*/plant, grey bars) or WDV (2 P*. alienus*/plant, white bars) for 24 hours IAP. Twenty-one days after inoculation, the sanitary status of each plant was tested by DAS-ELISA against BYDV-PAV and WDV for the calculation of the infection rates (*I_Si_*). Experiments were carried out three times with series of 20 plants/virus species/DAI/replicate. Histogram bars and error bars represent mean and standard error of *I_Si_*, respectively. Letters (a and b) were obtained after statistical analysis of the data (GLM (family binomial) followed by EMM *post-hoc* test). For each virus species (in bold for WDV), different letters indicate a significant difference (α = 0.05) between plant age at the inoculation.

Insect-mediated inoculation (2 insects/plant and 24 hours inoculation access period (IAP)) of either BYDV-PAV (transmitted by *R. padi*) or WDV (transmitted by *P. alienus*) were carried out on wheat plantlets of different ages (i.e. 7-, 9-, 12-, 15- and 19-days old) to calculate infection rates for each virus under single infection procedures (*I_Si_*, **Table 1**). Under our experimental procedures, BYDV-PAV and WDV isolates infected 80.0% (± 5.8%) and 83.3% (± 6.7%) of 7-days old inoculated plants, respectively (*I_Si_*; **Figure 1**). For BYDV-PAV, infection rates reached 88.3% ± 7.3% for 9-days old inoculated plants and decreased for older plants at inoculation (12- 15- and 19-days old plants were infected at a rate of 64.8% (± 11.5%), 36.2% (± 7.7%) and 52.7% (± 1.5%), respectively, **Figure 1**). This pattern of infection rates for different ages of inoculated plants was also observed for WDV (infection rates ranged from 92.8% (± 3.7%) to 59.3% (± 5.4%) for inoculation carried out on 9- to 19-days old plantlets). The infections rates of WDV did not vary with plant age at inoculation (GLM; *P*_WDV_= 0.11). In contrast, plant age at inoculation impacted significantly the *I_Si_* of BYDV-PAV (GLM; *P*_BYDV-PAV_= 6.0 x 10^-9^), suggesting that infection rate of this virus were lower on 12- and 15 days-old plants at inoculation compared to observed with 7-, 9-days old (Tukey; *P*< 0.05) (**Figure 1**).

#### Accumulation of BYDV-PAV and WDV in singly infected plants

**Figure 2:**
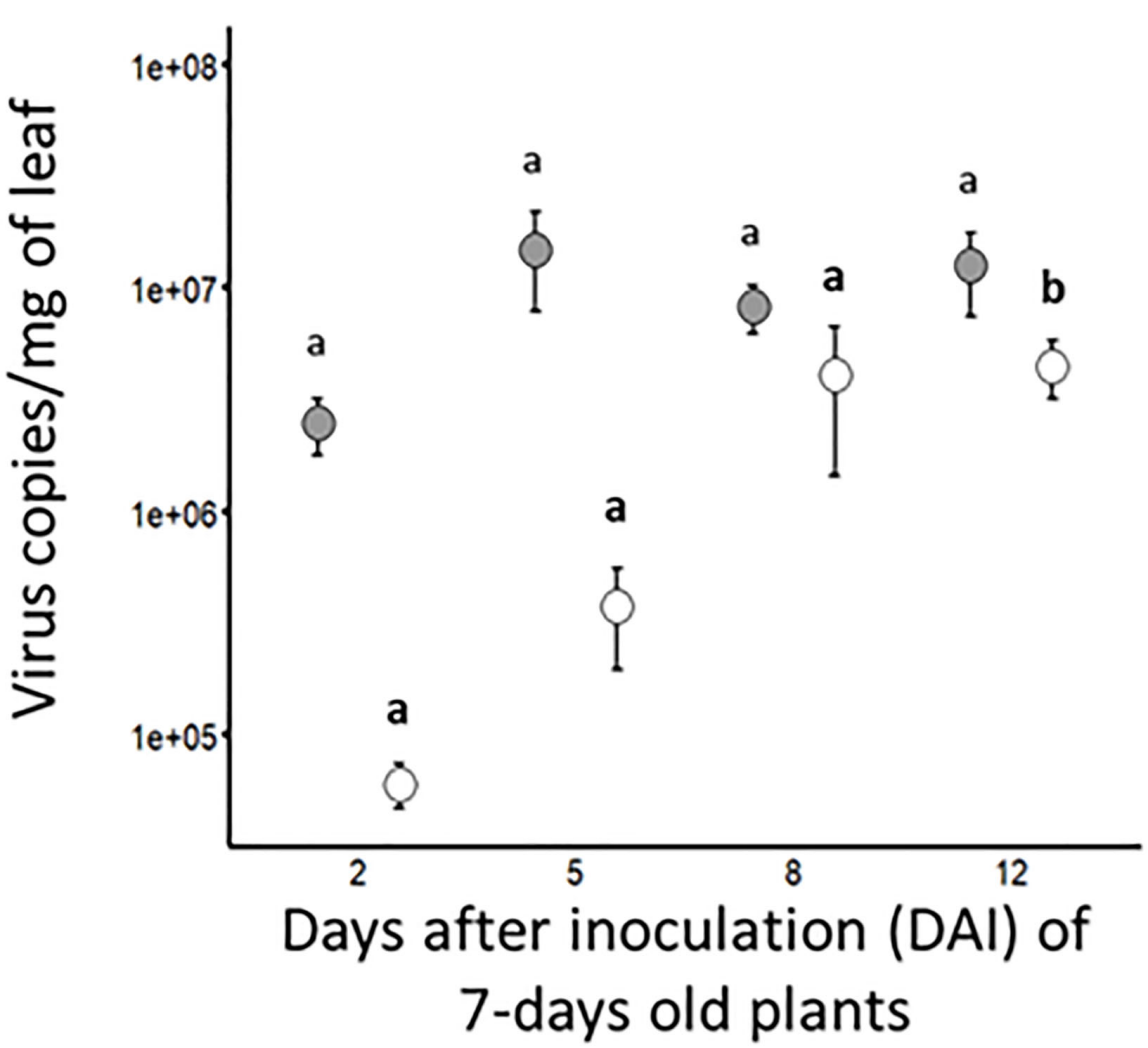
Kinetic of viral accumulations in singly infected plants. At different dates after inoculation (i.e. 2, 5, 8 and 12 DAI) of wheat plants (7-days old plants at inoculation; 5 viruliferous *R. padi* or *P. alienus* for 24 h IAP) with BYDV-PAV (in grey) and WDV (in white), viral copies/mg of leaf was calculated by (RT)-qPCR. Points and error bars represent mean and standard error of the viral loads, respectively. These two parameters were calculated using infected plants (BYDV-PAV; 2 DAI: *n*= 10, 5 DAI: *n*= 13, 8 DAI: *n*= 22 and 12 DAI: *n*= 22; WDV; 2 DAI: *n*=5, 5 DAI: *n*= 11, 8 DAI: *n*= 31 and 12 DAI: *n*= 31) obtained after three repetitions of the experimental design. Letters (a and b) were obtained after statistical analysis of the data (GLM (family binomial) followed by EMM *post-hoc* test). For each virus species (in bold for WDV), different letters indicate a significant difference (α = 0.05) between DAI.

Viral accumulation was evaluated by RT-qPCR (for BYDV-PAV) and qPCR (for WDV) at different days after the inoculation (DAI) of 7-days old wheat plants (i.e. at 2, 5, 8 and 12 DAI, **Figure 2** and **Table 3**). At 2 DAI, the titer of BYDV-PAV genome in infected plants reached 2.5 x 10^6^ (± 7.2 x 10^5^) copies/mg of leaf (*n*= 10). BYDV accumulation in infected plant varied slightly (from 2.5 x 10^6^ (± 7.2 x 10^5^) to 1.5 x 10^7^ (± 7.0 x 10^6^) BYDV-PAV copies/mg of leaf) with DAI (5 DAI (*n*= 13); 8 DAI (*n*= 22) and 12 DAI (*n*= 22)) reaching, during the kinetics of virus accumulation, the maximum viral load at 5 DAI (**Figure 2** and **Table 3** Singly infected plants). WDV titer tended to increase over time (2 DAI (*n*= 5); 5 DAI (*n*= 11); 8 DAI (*n*= 31) and 12 DAI (*n* = 31)), reaching maximal virus concentration in plants at 12 DAI (4.5 x 10^6^ (± 1.3 x 10^6^); **Figure 2** and **Table 3**). Statistical analysis showed that BYDV-PAV titer did not vary with DAI (ANOVA; *P*= 0.92). In contrast, WDV titer was significantly impacted by DAI (ANOVA; *P* = 2.11 x 10^-5^). Higher WDV titer was measured at 12 DAI compared to 2, 5, and 8 DAI (Tukey; *P* ≤ 0.05).

**Table 3:** BYDV-PAV and WDV loads in singly and co-infected plants at different days of infection.

DoI^*^	BYDV-PAV copies/mg of leaf								WDV copies/mg of leaf							
	Singly infected plants^†^				Co-infected plants				Singly infected plants^†^				Co-infected plants			
	Mean		SE^**^	*n*	Mean		SE^**^	*n*	Mean		SE^**^	*n*	Mean		SE^**^	*n*
2	2.5 x 10^6^ a	±	7.2 x 10^5^	10	2.1 x 10^6^ a	±	1.5 x10^6^	8	6.0 x 10^4^ **a**	±	1.3 x 10^4^	5	5,4 x 10^4^ **a**	±	1.8 x 10^4^	8
5	1.5 x 10^7^ a	±	7.0 x 10^6^	13	2.2 x10^7^ a	±	1.1 x 10^7^	15	3.8 x 10^5^ **a**	±	1.8 x 10^5^	8	2.7 x 10^5^ **a**	±	1.3 x 10^5^	15
8	8.2 x 10^6^ a	±	2.0 x 10^6^	22	3.7 x 10^7^ a	±	1.4 x 10^7^	20	4.1 x 10^6^ **a**	**±**	2.6 x 10^6^	31	4.4 x 10^5^ **a**	**±**	3.9 x 10^5^	20
12	1.3 x 10^7^ a	±	5.1 x 10^6^	22	1.3 x 10^7^ a	±	4.3 x 10^6^	21	4.5 x 10^6^ **a**	±	1.3 x 10^6^	31	7.1 x 10^6^ **a**	±	3.9 x 10^6^	21

^*^: Days after the inoculation of 7-days old plants.

^†^: These data correspond to **Figure 2**.

^**^: Standard error.

For a virus species, letters (in bold for WDV) illustrate the significant difference in virus load between singly and co-infected plants.

#### Virus transmission from singly infected plants

**Figure 3:**
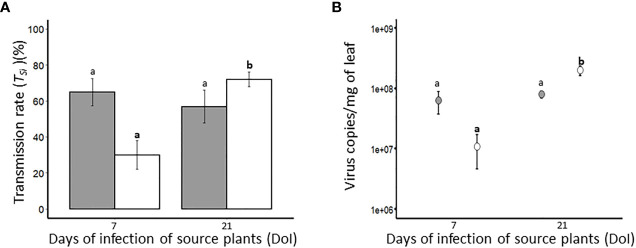
Transmission rate (*T_Si_*) **(A)** and viral load in BYDV-PAV and WDV source plants **(B)**. Plants (7-days old at inoculation; 5 viruliferous *R. padi* or *P. alienus* for 24 h IAP) infected with BYDV-PAV (in grey) or WDV (in white), were used as *source* plants in transmission experiments. At 7 and 21 days of infection (DoI), virus-free insects were deposited on infected *source* plants for AAP (BYDV-PAV: 6 h; WDV: 24 h) before being transferred on 7-days old *test* plants (1 *R. padi* or 2 P*. alienus* per *test* plant). For each source plant, the sanitary status of *test* plants was individually tested (3 weeks after inoculation) for the calculation of *T_Si_* **(A)**. In addition, *source* plants were sampled after each transmission experiment (at the end of AAP) and individually tested by (RT)-qPCR to evaluate BYDV-PAV and WDV viral loads **(B)**. Histogram bars **(A)** and points **(B)** represent mean of *T_si_* and viral load, respectively. Error bars represent standard errors. These parameters were calculated from infected plants (BYDV-PAV; 7 DAI: *n*= 20, 21 DAI: *n*= 16; WDV; 7 DAI: *n*= 16, 21 DAI: *n*= 21) obtained after three repetitions of the experimental design. Letters (a and b) were obtained after statistical analysis of the data (GLM (family binomial) followed by EMM *post-hoc* test). For each virus species (in bold for WDV), different letters indicate a significant difference (α = 0.05) between DoI.

Transmission experiments were carried out using virus-free vectors to evaluate the transmission efficiency of BYDV-PAV (1 *R. padi*/plant; 6 hours acquisition access period (AAP)) and WDV (2 P*. alienus*/plant; 24 hours AAP) from source plants inoculated at 7-days old. After 7 (n= 20) and 21 (n= 16) days of infection of source plants (DoI), the transmission efficiency of BYDV-PAV was 64.9% (± 7.5%) and 56.9% (± 9.0%) (*T_Si_*, **Table 1**), respectively (**Figure 3A**). For WDV, transmission rate increased between the 7^th^ (29.9% (± 8.0%); *n*= 16) and 21^st^ (71.9% (± 4.1%); *n*= 21) day of infection of WDV source plants (**Figure 3A**). Transmission rates of BYDV-PAV calculated using this experimental procedure were not impacted by DoI (GLM; *P*= 0.5). In contrast, DoI significantly impacted the *T_Si_* of WDV (GLM; *P*= 1.5 x 10^-05^), indicating that the transmission rate of WDV from source plants at 7 DoI was lower than from source plant at 21 DoI (Tukey; *P* ≤ 0.05). Source plants (at 7 and 21 DoI) used for transmission experiments were individually sampled and tested (using (RT)-qPCR approach) for virus concentration to assess whether the observed variations of the transmission efficiency can be associated with changes in virus accumulation in source plants (**Figure 3B** and **Table 4**). The accumulation of WDV varied significantly with DoI of source plants (ANOVA; *P*= 1.4 x 10^-5^), whereas, the accumulation of BYDV-PAV did not (ANOVA; *P*= 0.53).ANOVA; *P*=0.001). WDV accumulation in source plants inoculated for 7 days was lower than in source plants at 21 DoI (Tukey; *P* ≤ 0.05).

**Table 4:** Virus load in singly and co-infected source plants at 7 and 21 days of infection.

DoI^*^	BYDV-PAV copies/mg of leaf								WDV copies/mg of leaf							
	Singly infected plants^†^				Co-infected plants				Singly infected plants^†^				Co-infected plants			
	Mean		SE**	*n*	Mean		SE^**^	*n*	Mean		SE^**^	*n*	Mean		SE^**^	*n*
7	6.4 x 10^7^ a	±	2,6 x 10^7^	18	7.8 x 10^7^ a	±	2.3 x10^7^	21	1.1 x 10^7^ **a**	±	6.3 x 10^6^	18	1.9 x 10^6^ **a**	±	1.17 x 10^6^	10
21	8.0 x 10^7^ a	±	1.1 x 10^7^	17	8.0 x10^7^ a	±	7.3 x 10^6^	21	2.0 x 10^8^ **b**	±	1.8 x 10^8^	21	8.5 x 10^7^ **a**	±	1.1 x 10^7^	20

^*^: Days of infection of source plants.

^†^: Data illustrated in **Figure 3B**.

^**^: Standard error.

For a virus species, letters (in bold for WDV) illustrate a significant difference in viral load between singly and co-infected plants.

### Simultaneous inoculations: co-infection and virus transmission

#### Impact of plant age on co-infection rate

**Figure 4:**
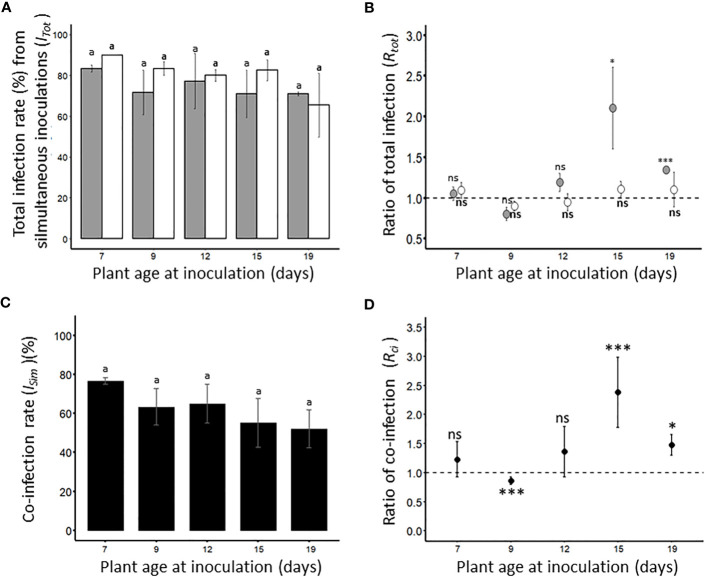
Total infection rate of viruses **(A, B)** and occurrence of co-infections **(C, D)** after simultaneous inoculations of BYDV-PAV and WDV. Seven-days old wheat plants were simultaneously inoculated (2 viruliferous *R. padi* and 2 viruliferous *P. alienus* for 24 h IAP) with BYDV-PAV and WDV. Twenty-one days after inoculation, the sanitary status of each plant was tested by DAS-ELISA against the two viruses. Experiments were carried out with series of 20 plants/virus species/DAI and repeated three times. For each virus species/plant age at inoculation combination, the total infection rate of BYDV-PAV (in grey) and WDV (in white) was calculated **(A)**. Then, these data were normalized by infection rates obtained after the singly inoculation of each virus (see. **Figure 1**) to calculate the ratio of total infection (*R_tot_*) **(B)**. Co-infection rate (*I_Sim_*, in black) was calculated for each virus species/plant age combination **(C)** and divided by the theoretical co-infection rate to obtain the ratio of co-infection (*R_ci_*) **(D)**. Histogram bars **(A, C)** and points **(B, D)** represent the mean of monitored values. Errors bars represent the standard errors. Letters (i.e. a) and asterisks (ns: non significant, *: P≤ 0.05, **: P≤ 0.01, ***: P≤ 0.001) were obtained after statistical analysis (GLM (family binomial) followed by EMM *post-hoc* test **(A, C)**, or χ² test **(D)**) of raw data. For a virus species (in bold for WDV), these symbols illustrate a significant/non-significant difference (α = 0.05) between i) plant age **(A, C)**, ii) the type of inoculation (i.e. single versus simultaneous inoculation) **(B)** or iii) observed and theoretical co-infection rates **(D)**.

To test whether simultaneous inoculation of BYDV-PAV and WDV could alter the ability of these two cereal viruses to infect susceptible wheat, co-inoculations were carried out on 7-, 9-, 12-, 15- and 19-days old plants. Under our experimental conditions, the total infection rate of each virus after simultaneous inoculations (*I_Tot_*, **Table 1**) was calculated (**Figure 4A**). The *I_Tot_* values obtained for either BYDV-PAV or WDV did not vary with plant age at inoculation (GLM; *P_BYDV-PAV_* = 0.81; *P_WDV_* = 0.22). To assess whether simultaneous inoculations increased the ability of BYDV-PAV and WDV to infect wheat, *I_Tot_* was normalized by *I_Si_* (see **Figure 1**) to calculate the ratio of total infection (*R_tot=_I_Tot_/I_Si_*, **Table 2**. 4B). For BYDV-PAV, *R_tot_* values were i) close to 1 for 7- (*R_tot_D7 =* 1.1 ± 0.1), 9- (*R_tot_D9 =* 0.8 ± 0.1) and 12- days (*R_tot_D12 =* 1.2 ± 0.1) old inoculated plants, and ii) above 1 for wheat plants aged of 15 (*R_tot_D15 =* 2.1 ± 0.5) and 19 (*R_tot_D19 =* 1.3 ± 0.04) days at inoculation (**Figure 4B**). The *R_tot_* values obtained for WDV were close to 1 for each tested plant age at inoculation (*R_tot_D7 =* 1.1 ± 0.1; *R_tot_D9 =* 0.9 ± 0.1; *R_tot_D12 =* 1.0 ± 0.1; *R_tot_D15 =* 1.1 ± 0.1; *R_tot_D19 =* 1.1 ± 0.2) (**Figure 4B**). For wheat plants inoculated at 7-, 9- and 12- days old, data showed that the *I_Tot_* of BYDV-PAV and WDV are similar to *I_Si_* of the corresponding viral species (GLM; BYDV-PAV: *P_D7 =_* 0.57, *P_D9 =_* 0.19, *P_D12 =_* 0.59; WDV: *P_D7 =_* 0.27, *P_D9 =_* 0.10, *P_D12 =_* 0.57). *I_Tot_* values of BYDV-PAV were significantly higher than *I_Si_* for 15- (GLM; *P*= 0.03) and 19-days (GLM; *P*= 2.2 x 10^-16^) old wheat plants, whereas infection rates of WDV obtained from plants inoculated at 15- and 19-days old were not impacted by simultaneous inoculations procedures (GLM; *P_D15 =_* 0.40, *P_D19 =_* 0.48). The impact of simultaneous inoculations on the occurrence of co-infection was analysed. Co-infection rates (*I_Sim_*, **Table 1**) between the two viruses varied from 76.67% (± 1.66%) (*I_Sim_ D*_7_) to 52.1% (± 9.8%) (*I_Sim_ D*_19_) depending on the age of plant at the inoculation (**Figure 4C**). The co-infection rates observed for the different ages of plant at inoculation are not significantly different (GLM, *P*= 0.12). The *I_Si_* of BYDV-PAV and WDV can be used to calculate theorical co-infection rates (*I_Th_*, **Table 1**). Then, *I_Th_* values were used to calculate co-infection ratios (*R_ci_=I_Sim_/I_Th_*; **Table 2**; **Figure 4D**). Except for *R_ci_ D*_9_ (0.8 ± 0.01), *R_ci_* reached values above 1 (*R_ci_ D_7 =_* 1.2 ± 0.2, *R_ci_ D_12 =_* 1.2 ± 0.3, *R_ci_ D_15 =_* 2.1± 0.5 and *R_ci_ D_19 =_* 1.7 ± 0.2). Statistical analysis of data showed that the proportion of co-infected plants obtained with plants inoculated at 7-and 12-days old were similar to the corresponding theorical co-infection rates (χ²; *P_D7 =_* 0.20, *P _D12 =_* 0.28, respectively). On plants inoculated at 9 days old, *I_Sim_ D*_9_ co-infection was significantly lower than *I_Th_* (χ²; *P*= 7.71 x 10^-5^), while co-infections were observed with significant higher frequencies on plants inoculated at 15-and 19-days old (χ²; *P_D15 =_* 3.91 x 10^-8^, *P_D19 =_* 0.01, respectively).

#### Virus accumulation in co-infected plants

BYDV-PAV and WDV load in co-infected plants was measured by (RT)-qPCR at different DAI of 7-days old inoculated plantlets (i.e. at 2, 5, 8 and 12 DAI; **Table 3**, Co-infected plants). These data were compared with virus accumulation in singly infected plants (**Table 3**, Singly infected plants and **Figure 2**). BYDV-PAV and WDV seemed to accumulate higher (ANOVA; *P*= 0.02; **Supplementary Figure 1.A**) and lower (ANOVA; *P*= 0.01; **Supplementary Figure 1.B**), respectively, in co-infected wheat plantlets than in singly infected plants. However, *post-hoc* analysis (i.e. Tukey test) of virus concentrations in infected plants at each DAI showed that BYDV-PAV and WDV accumulated similarly between singly and co-infected plants from the 2^nd^ to the 12^th^ days of virus infection (**Table 3**).

#### Transmission of viruses from co-infected plants

**Figure 5:**
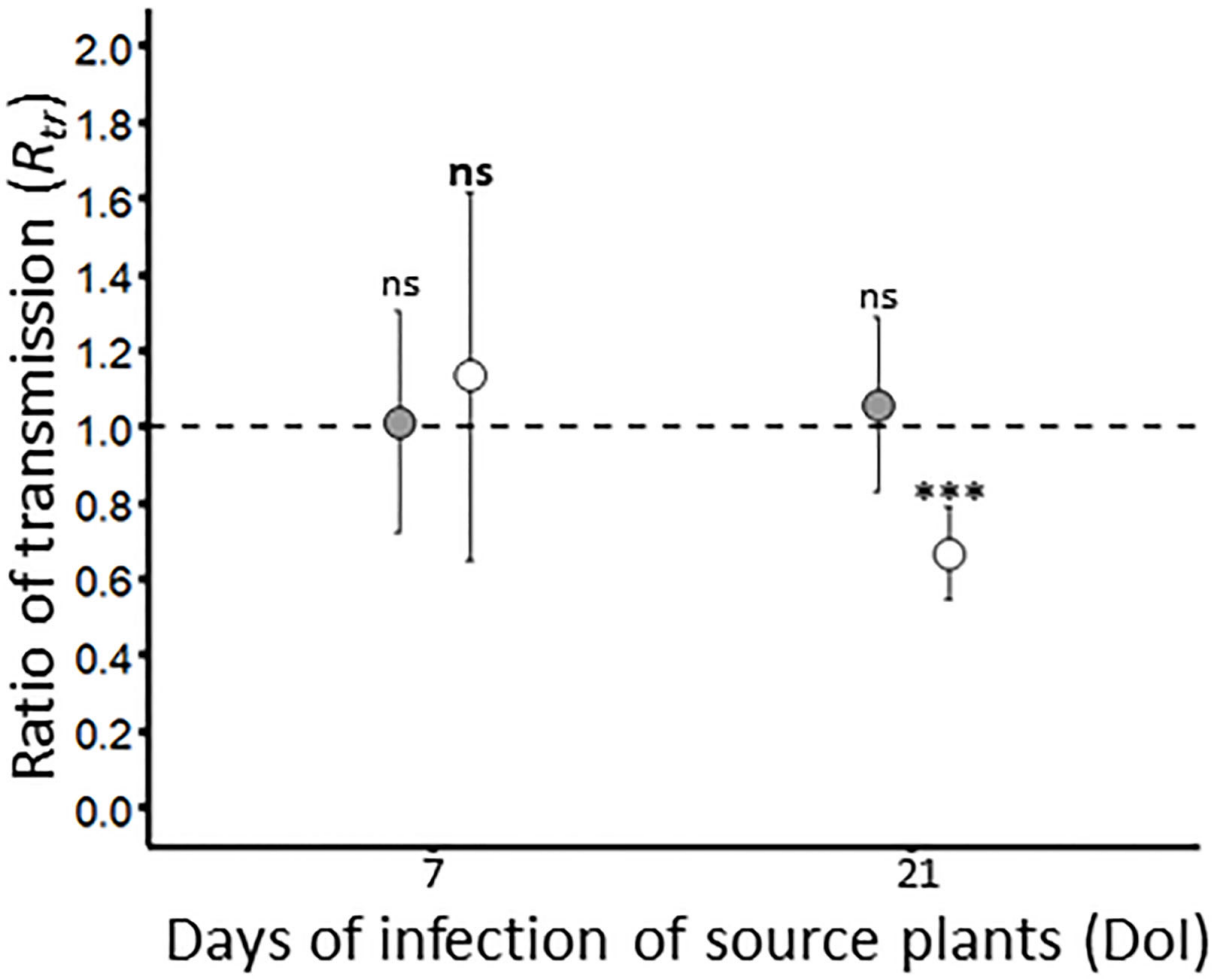
Ratio of transmission of BYDV-PAV and WDV. By combining the infection rates obtained with singly (see **Figure 3A**) and co-infected (**Supplementary Figure 2**) source plants, the ratio of transmission *R_tr_* was calculated for BYDV-PAV (in grey) and WDV (in white). Points and error bars represent mean and standard errors of the *R_tr_*, respectively. These two parameters were calculated on three repetitions of the experimental design. ‘ns’ and ‘***’ were obtained after statistical analysis (GLM (family binomial)) carried out on the raw data. For a virus species/DoI combination (in bold for WDV), ‘***’ illustrates the significance difference (P≤ 0.001) between singly and co-infected plants. ns: non-significant.

The impact of co-infection of plants with BYDV-PAV and WDV on the plant-to-plant transfer of these viruses was evaluated. Seven days old plantlets were simultaneously inoculated and used as source of viruses to carry out transmission experiments. The transmission rates of BYDV-PAV from co-infected source plants reached 51.5% ± 9.1% and 55.7% ± 5.5% using source plants at 7 and 21 DoI, respectively (*T_Ci_*, **Table 1** and **Supplementary Figure 2**). WDV was transmitted at *T_Ci_* of 40.2% ± 7.5% and 44.4% ± 5.3% with source plants at 7 and 21 DoI, respectively (**Supplementary Figure 2**). Based on these observations, transmission rates of BYDV-PAV and WDV did not vary with DoI of co-infected source plants (GLM; DoI: *P_BYDV-PAV_=* 0.67; *P_WDV_ =* 0.52). To compare these data to the transmission rate from singly infected plants (*T_Si_*; see **Figure 3A**), transmission ratio (*R_tr_*, **Table 2**) was built for each virus/DoI combination (**Figure 5**). *R*_tr_ values were close to 1, except for WDV transmission rate at 21 DoI (*R*_tr_= 0.7 ± 0.1). Data analysis showed that transmission rates from source plants used at 7 DoI vary with experimental repetitions (*P_BYDV-PAV_=* 0.02; *P_WDV_=* 9.0 x 10^-5^). A GLM analysis carried out with “experimental repetitions”, “co-infection” and “interaction” as factors showed that source plants (i.e. singly and co-infected) used for BYDV-PAV transmission at 7 and 21 DoI did not vary with co-infection (*P_7DoI_=* 0.24; *P_21DoI_=* 0.91) and its interaction with experimental repetitions (*P_7DoI_=* 0.12; *P_21DoI_=* 0.45). Similar results were obtained for WDV transmission from WDV and co-infected source plants at 7 DoI (GLM; co-infection: *P=* 0.21; interaction: *P=* 0.49). However, lower transmission rates of WDV were reported from co-infected source plants (GLM; *P=* 1.3 x 10^-5^). Co-infection effect was not associated with experimental repetitions (GLM; experimental repetition: *P*= 0.13; interaction: *P=* 0.11). This validates the negative effect of co-infection on the transmission efficiency of WDV observed at 21 DoI (**Figure 5**). To complete this analysis, BYDV-PAV and WDV virus loads were measured in co-infected source plants used in transmission experiments (**Table 4**). When compared with source plants singly infected with BYDV-PAV (see **Figure 3B** and **Table 4**), similar loads of BYDV-PAV were measured on singly and co-infected source plants at 7 and 21 DoI (ANOVA; co-infected source plants: *P*= 0.68; DoI: *P*= 0.59; interaction: *P*= 0.69; **Table 4**). WDV loads varied significantly between singly and co-infected plants (ANOVA; *P*= 0.02), the DoI (ANOVA; *P*= 9.7 x 10^-8^) and with the interaction of these factors (ANOVA; *P*= 0.03). No significant variation of WDV loads were observed in singly and co-infected source plants at 7 DoI, whereas at 21 DoI WDV accumulates lower (8.5 x 10^7^ (± 1.1 x 10^7^)) in co-infected plants compared with singly infected source plants (2.0 x 10^8^ (± 1.8 x 10^8^)) (Tukey; *P* ≤ 0.05).

### Sequential inoculations: impact of pre-infections

Wheat plantlets (7-days old wheat plants) were mock inoculated with virus-free *P. alienus* or virus-free *R. padi.* At several timepoints, i.e. 2, 5, 9 and 12 days after mock inoculation (DaMI), plants were inoculated with vectors collected from infected rearing systems. This experiment showed that infection rates of BYDV-PAV and WDV were not impacted by mock inoculations (GLM; *P_BYDV_* = 0.56; *P_WDV_* = 0.08) regardless the age of plant at inoculation (GLM; interaction; *P_BYDV_* = 0.21; *P_WDV_* = 0.74) (**Table 5**).

**Table 5:** Infection rate of BYDV-PAV and WDV after mock inoculation.

DaMI*	Mock inoculation	Infection rate (*I_Si_*) (%) (Mean ± SE**)						
		BYDV-PAV			WDV			
2	No^†^	88.33 a	±	7.26	92.75 **a**	±	3.66
	Yes	78.04 a	±	8.54	81.08 **a**	±	8.06
5	No^†^	64.90 ab	±	11.55	86.57 **a**	±	11.03
	Yes	65.78 ab	±	10.99	66.57 **a**	±	18.79
9	No^†^	36.18 b	±	7.66	76.57 **a**	±	7.34
	Yes	49.61 b	±	5.44	55.10 **a**	±	13.15
12	No^†^	52.65 ab	±	1.45	59.31 **a**	±	5.38
	Yes	61.86 ab	±	4.60	57.92 **a**	±	14.07

^*^: Days of infection of source plants.

^†^: Data illustrated in **Figure 3B**.

^**^: Standard error.

For a virus species, letters (in bold for WDV) illustrate a significant difference in viral load between singly and co-infected plants.

**Figure 6:**
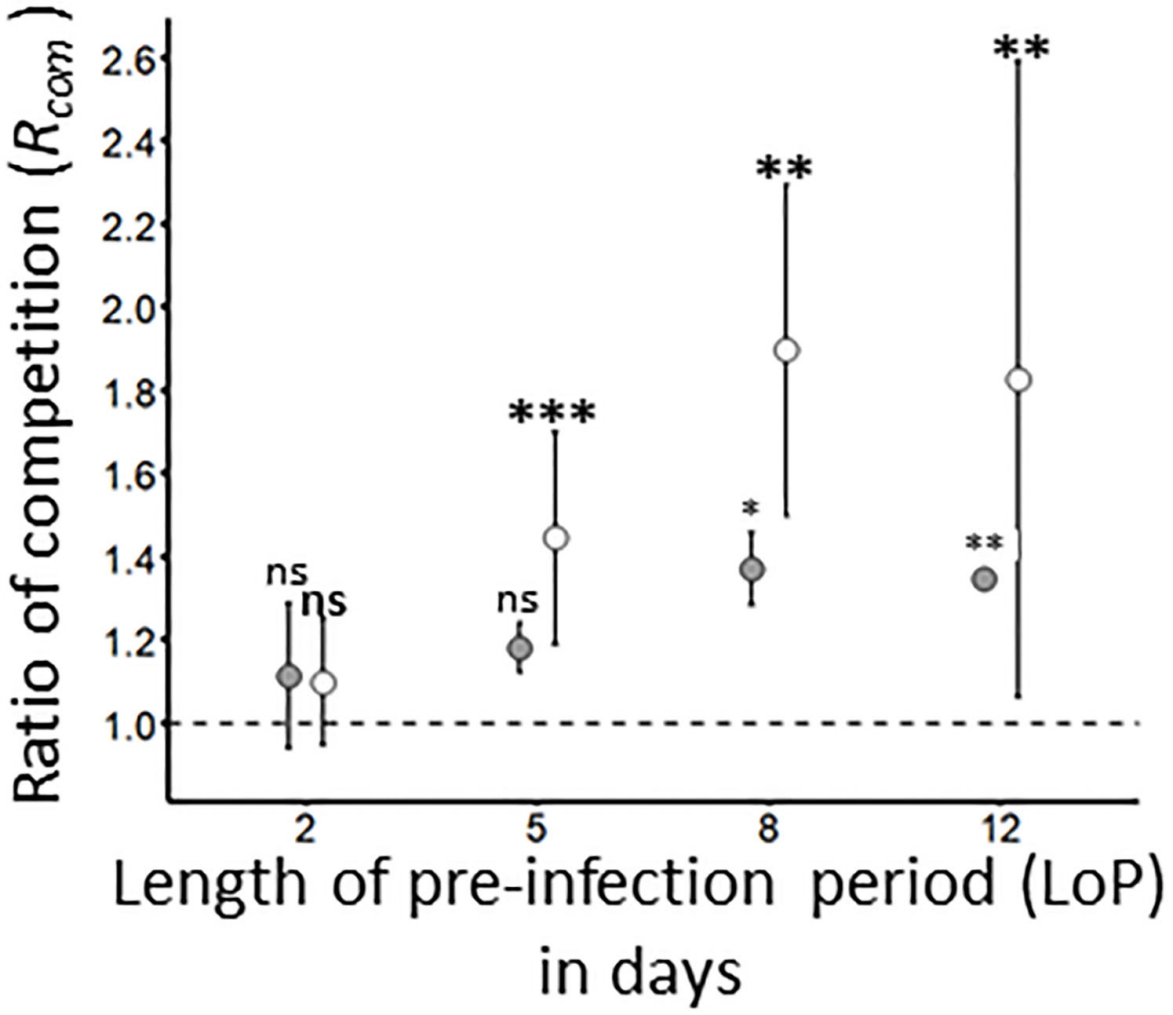
Ratio of competition for BYDV-PAV and WDV on pre-infected plants. By combining the data obtained with mock-inoculated (see **Table 5**) and pre-infected plants (**Supplementary Figure 3**), the ratio of competition *R_com_* was calculated for WDV – BYDV-PAV (W/B; in grey) and BYDV-PAV – WDV (B/W; in white) sequential inoculations carried out with different lengths (from 2 to 12 days) of pre-infection (LoP). Points and error bars represent mean and standard errors associated to *R_com_* values, respectively. The *R_com_* was calculated for three repetitions of the experimental design. ‘ns’, ‘*’, ‘**’ and ‘***’ were obtained after statistical analysis (GLM (family binomial)) carried out on raw data. For a treatment/LoP combination (in bold for B/W treatment), asterisks illustrate significant differences (*: P≤ 0.05, **: P≤ 0.01, ***: P≤ 0.001) between mock-inoculated and pre-infected plants. ns: non-significant.

To assess whether a pre-infection of wheat plant could impact the inoculation of a competitor virus, sequential inoculations were carried out using a 2-, 5-, 8- and 12-days period between the two inoculations steps. Under these experimental conditions, co-infection rate (*I_Seq_*, **Table 1**) of i) BYDV-PAV on WDV pre-infected plants (_W/B_) and ii) WDV on BYDV-PAV pre-infected plants (_B/W_) did not vary significantly with the length of the pre-infection period (LoP) (GLM; *P*_B/W_= 0.16; *P*_W/B_= 0.24; **Supplementary Figure 3**). For each virus/(DaMI or LoP) combination, the competition ration (*R_com_*, **Table 2** and **Figure 6**) was built by normalizing the *I_Seq_* (**Supplementary Figure 3**) with the infection rate obtained from single inoculation of mock-inoculated plants (see **Table 5**). The *R_com_* of _W/B_ increased progressively (*R_com_ LoP*_2 =_ 1.1 ± 0.2; *R_com_ LoP*_5 =_ 1.2 ± 0.1) to reach maximal value at LoP_8_ and LoP_12_ (*R_com_ LoP_8 =_ 1*.4 ± 0.02; *R_com_ LoP_12_*: 1.4 ± 0.1). The *R_com_* of _B/W_ presents a similar trend (*R_com_ LoP_2 =_* 1.1 ± 0.2; *R_com_ LoP_5 =_* 1.5 ± 0.3; *R_com_ LoP_8 =_* 1.9 ± 0.4; *R_com_ LoP_12 =_* 1.8 ± 0.8). However, values associated to LoP from _B/W_ experiment were higher than *R_com_* values obtained from _W/B_ inoculation procedure (**Figure 6**). Data analysis carried out on raw data showed that pre-infection of 2 days had no impact on the infection success of BYDV-PAV and WDV (GLM; LoP_2_: *P*_W/B_ = 0.62; *P*_B/W_= 0.64). After 5 days of pre-infection, similar results were obtained for _W/B_ (GLM; *P*_W/B_= 0.51), whereas, the infection success of _B/W_ plants was significantly higher than infection rate obtained from _M/W_ (*R. padi-*mock/WDV inoculated plants; GLM; *P*_B/W_ LoP_5 =_ 8.1 x10^-5^)). Finally, after 8 and 12 days of pre-infection, a significant increase of infection success is observed for _W/B_ and _B/W_ (GLM; LoP_8_: *P*_W/B_= 0.01, *P*_B/W_= 0.001; LoP_12_: *P*_W/B_= 0.005, *P_(_*_B/W_= 0.006).

## Discussion

Analyses of the epidemiology of plant viral diseases have long been based on data collected from biological, evolutionary and ecological properties of each partner involved in studied pathosystems. Co-infection of hosts, frequently observed in cultivated and uncultivated areas (Zinga et al., 2013; Jarošová et al., 2018; Liu et al., 2020), can change nature and intensity of the interactions between viruses, vectors and hosts, and thus modify our understanding of ecology and epidemiology of plant viruses (Alcaide et al., 2020). Plants co-infected with BYDV-PAV and WDV have been reported in the literature. However, little is known about if and how the sympatry between these two viruses can benefit or constrain their epidemiology. In this study, we evaluated the impact of co-infections on parameters of the epidemiology of YDD and WDD diseases. Experiments were carried out to evaluate and compare, through different competition scenarios (i.e. single- and co- (simultaneous and sequential) inoculations), efficiency of BYDV-PAV and WDV to infect, to accumulate in and to be spread by their respective insect vector from wheat plants. This deciphering of the epidemiological processes associated with these two viral diseases allowed to accurately describe that BYDV-PAV and WDV achieve their infection cycle and their plant-to-plant transmission with different efficiencies, and interact asymmetrically during co-infection of wheat. Moreover, the impact of competition scenarios on biological parameters of these two viruses was evaluated at different stages of the infection cycle and using plants at different ages. Results showed that BYDV-PAV – WDV interactions lead to different phenotypes ranging from synergism (**Figure 4B**, **Figure 4D**, **Figure 6** and **supplementary Figure 1A**) to antagonism (**Figure 4D**, **Figure 5**, **Table 4** and **supplementary Figure 1A**).

For insect-borne viral diseases, the epidemiological process begins with the migration of viruliferous vector(s) from infected reservoirs towards crop fields. The intensity of primary inoculations relies on several parameters including abundance, sanitary status and behaviour (e.g. feeding and preferences) of insect vectors, and on the infection rate associated to virus inoculation (Jones et al., 2010). In western Europe, with winter wheat sown from early September to late October, YDD and WDD introductions in fields mainly occur in autumn. The aphid *R. padi* is the most abundant vector of BYDV-PAV in cereal fields in autumn (Gillet et al., 1990). The abundance and the population dynamics of this aphid species have been shown to predict YDD risk (Fabre et al., 2003a). The leafhopper *P. alienus*, the vector of WDV, is observed in winter cereal fields from sowing to the end of the year when temperatures are cold (Mehner et al., 2002). It has been shown that the abundance *R. padi* and *P. alienus* and the proportion of viruliferous individuals vary with space and time and are linked to the composition of the agroecosystem (Östman et al., 2001; Lindblad and Arenö, 2002; Mehner et al., 2002; Fabre et al., 2005). Insect behaviour (e.g. feeding, settling and mobility) is an important parameter determining which plants and how many plants are visited by each individual vector. Numerous factors, such as i) the plant species/cultivars (Tholt et al., 2015) and/or of competitor insect species (Alla et al., 2001), can impact preferences and/or feeding of insects. Moreover, it has been shown that behaviour traits of insects can be also influenced by their sanitary status and the sanitary status of visited host plants (Mauck et al., 2018). Finally, insects are able to re-take off several times after first landing leading to inoculation of several plants by a single insect (Parry, 2013; Abt and Jacquot, 2015). The abundance, the sanitary status and/or the behaviour of vectors have been extensively monitored and incorporated in predictive modelling studies targeting persistently transmitted plant viruses (Jones et al., 2010). For these viruses, the infection rate introduced in models is frequently considered as a constant close to 100% ((Jones et al., 2010) but see Thackray et al., 2009 for exception). However, under our experimental conditions infection rates of BYDV-PAV and WDV in a susceptible cultivar were i) below 93% and ii) can decreased with plant age at inoculation (e.g. BYDV-PAV; from 7 to 19 days old inoculated plants). These results suggest that during the first weeks after sowing, wheat plants show age-dependent level of susceptibility to these two viruses. This may lead to a range of efficiencies for virus introduction in wheat fields. Positive (Zhang et al., 2021) and negative (Lindblad and Sigvald, 2004) impacts of plant age on host susceptibility to pathogens have been reported for several pathosystems, suggesting that host plant development at inoculation represent a key parameter to understand the ecology and epidemiology of viral diseases (Ashby and Bruns, 2018). Together with these previously published works, our results highlight that, when modelling the intensity of the introduction of a given viral disease in fields, infection rate should not be considered as a constant either for a group of viruses (e.g. persistently transmitted viruses) or for a virus species (e.g. BYDV-PAV and WDV).

From sowing to harvest, several species of insects and/or viruses are introduced in and spread within fields. This continuous biological process leads to frequent virus co-inoculations of plants (Zinga et al., 2013; Tollenaere et al., 2016). The ecology of *P. alienus* (vector of WDV) and *R. padi* (vector of BYDV-PAV), and the report of cereal plants co-infected with BYDV-PAV and WDV (Jarošová et al., 2018; Liu et al., 2020) imply that these vectors occur in sympatry in cereal fields. However, under laboratory conditions, Alla et al. (2001) showed that simultaneous deposition on cereal plantlet of *R. padi* and *P. alienus* i) increased mortality, ii) slowed down the development and iii) modified the behaviour of the leafhopper *P. alienus*. These data are in favor to antagonistic interactions between these two insects. However, simultaneous inoculations of BYDV-PAV and WDV carried out on wheat plants of different ages (this work) is associated with an increase of the total infection rate of BYDV-PAV for 15-and-19 days old inoculated plants indicating that BYDV-PAV and WDV interact synergistically in favor to BYDV-PAV. Moreover, simultaneous inoculations led to either similar (i.e. 7-and 12-days old plants), lower (i.e. 9 days old plants) or higher (i.e. 15-and 19-days old plants) occurrence of co-infections compared with expectations from independency between these partners. This indicates that, depending on the age of inoculated plants, BYDV-PAV – WDV interactions resulting from simultaneous inoculations vary from antagonism to synergism, but the effect of these interactions on the total infection rate of viruses only benefit to BYDV-PAV.

For two insect-borne viruses transmitted by two different vectors, simultaneous inoculations require that insects land and inoculate viruses on the same plant in a short time window, which limit the probability of occurrence in fields. However, co-infections can also result from sequential inoculations of the two viruses. In that scenario, the pre-infecting virus has the opportunity to systemically infect the host which may impact the physiology of the latter (Alcaide et al., 2022). Then, compared with a healthy host, a pre-infected plant may constitute a different environment for a competitor virus modifying its capacity to replicate and/or to accumulate (Chávez-Calvillo et al., 2016). Depending on the delay between the two inoculation steps, pre-infection has neutral to beneficial effects on the infection rates of BYDV-PAV and WDV. Improvement of the infection rate of WDV occurred earlier than for BYDV-PAV, suggesting that the synergy between these viruses is asymmetric in favor to WDV. This indicates, as reported for other pathosystems (Alcaide et al., 2020; Moreno and López-Moya, 2020), that the order of inoculation of these two viruses is an important factor for virus-virus interactions in plant and their consequences on the epidemiology of associated diseases. Overall, our results show that the sympatry between BYDV-PAV and WDV impacts their infection rates in complex ways depending of plant age, pre-infection duration and the order of virus inoculation. The increase of BYDV-PAV infection rate resulting from simultaneous and sequential inoculations, suggests that the intensity of primary inoculations may be impacted by WDV. This could lead to more infected plants from which disease spread can occur within fields.

For WDV and BYDV-PAV, secondary inoculation is a key parameter for disease prevalence in cereals fields (Leclercq-Le Quillec et al., 2000; Lindblad and Sigvald, 2004). Thus, together with the dynamics of vector populations (Fabre et al., 2003a), agricultural practices (Östman et al., 2001; Lindblad and Waern, 2002) and the quality of reservoirs (Van den Eynde et al., 2020), the differences in accumulation of BYDV-PAV and WDV in infected plants could lead to contrasted efficiencies in plant-to-plant transmissions at field scale. While predictive modelling studies on viral disease epidemics are still lacking for WDV, several models exist for BYDV-PAV (Jones et al., 2010 and the reference therein). In these models, the latency period of the host (i.e. delay between infection and infectious status) are frequently considered as a constant varying from days (e.g. 4 days; Kendall et al., 1992) to weeks (e.g. 2.5 weeks; Thackray et al., 2009). Our data indicate that, in the susceptible wheat cv. Rubisko, plants infected with BYDV-PAV reached maximal infectious status before the 7^th^ day of infection, which is consistent with previously published data (Pichon et al., 2022). This result should be considered for any future improvement of BYDV-PAV epidemiological model.

The fitness of a virus *in planta* relies mainly on host-virus interactions. However, during co-infections, each virus uses host resources to complete its infectious cycle. This competitive phenomenon can modify the fitness landscape of each competitor (Elena et al., 2014). In this study, co-infections with BYDV-PAV and WDV led to opposite (i.e. positive and negative effects for BYDV-PAV and WDV, respectively) and antagonistic (i.e. against WDV) interactions. Mechanisms leading to changes in viral fitness during co-infections can result from indirect (i.e. involving host-virus interactions) and/or direct interactions between viruses (Tollenaere et al., 2016). Viral silencing is the main host defense mechanism involved in antagonistic and synergistic effects of co-infections (Moreno and López-Moya, 2020). Interactions between viral proteins may lead to negative or positive impact(s) in one or several step(s) (e.g. host range (Abt et al., 2020), replication (Fontenele et al., 2021) and or cellular tropism (Mayo et al., 2000)) of the virus infection cycle. Direct mechanisms require intimate relationship between viral component(s), which is more likely to result from long term and frequent sympatry between viruses. While BYDV-PAV and WDV have been reported in France for several decades, the frequency of co-infections of plants in wheat fields has not been accurately surveyed yet. However, the prevalence of WDV in French fields has been reported to be highly variable in both space and time (Abt and Jacquot, 2015), which seems to reduce the likelihood of frequent co-infection. In this scenario, indirect mechanism(s) may be more likely involved in the changes in the fitness in planta of these viruses during co-infections. This should be confirmed in future studies aiming at deciphering the molecular mechanisms underlying the interactions between BYDV-PAV and WDV in co-infected plants.

Transmission of BYDV-PAV was not impacted by co-infection with WDV. A similar result was found for WDV at early stage of infection, whereas the capacity of co-infected plants to be source of WDV for insect-mediated transmission was reduced at late stage of infection. This asymmetric antagonism is associated with lower WDV load in co-infected plants. These results are consistent with the accumulation-transmission trade off (Froissart et al., 2010) and with previous reports evaluating the effect of co-infections on the accumulation and transmission of other persistently transmitted viruses (e.g. Péréfarres et al., 2014). However, changes in the feeding behaviour of vectors on co-infected plants have also been reported in the literature (Domingo-Calap et al., 2020; Jia et al., 2022). Thus, modifications of *P. alienus* behaviour on BYDV-PAV – WDV co-infected plants should also be considered as a possible factor for the observed lower WDV transmission rate from co-infected source plants. As a change in the source quality of a host is expected to alter pathogen spread in a healthy host population (Froissart et al., 2010), our results suggest that co-infections may negatively impact the spread of WDV in wheat fields. However, virus spread also relies on the performances and behaviour of insect vectors (Shaw et al., 2017). These parameters can be modified by sanitary status of host plant (Domingo-Calap et al., 2020) and/or the presence of a competitor insects (Alla et al., 2001). To our knowledge, the effect of WDV – BYDV-PAV co-infections on vector traits have not yet been reported yet. In the future, studying how co-infections can impact the different steps involved in plant-to-plant transmission (i.e. acquisition, latency in vector and inoculation parameters) and vector traits may contribute to a better description of the consequences of the sympatry of BYDV-PAV and WDV on the sanitary status of cereal fields.

Numerous studies on viral co-infections have only evaluated viral fitness *in planta* and/or virulence under a low number of experimental conditions. These approaches do not allow *per se* to fully understand the effect of co-infections on the epidemiology on viral diseases. To better understand the impact of BYDV-PAV – WDV interactions on the epidemiology of these two viruses, we included several parameters involved in introduction, maintenance and spread of viruses in our experimental design. Our results suggest that the presence of WDV could have neutral to positive effects on biological parameters involved in primary (i.e. infection rate following simultaneous and sequential inoculations) and secondary (i.e. viral accumulation and transmission efficiency) inoculations of BYDV-PAV in wheat fields. Consequently, this suggests that the epidemiology of BYDV-PAV could benefit from the co-occurrence of WDV. For the latter, sequential inoculations with BYDV-PAV may improve the success of primary infections. However, co-infected plants are poor sources of WDV for leafhopper-mediated transmission, which could negatively impact the spread of this virus in a healthy plant population. These data highlights that the sympatry between BYDV-PAV and WDV seems to exert opposite pressures on WDV epidemiology, making it difficult to evaluate the net effect of co-infections on the epidemiology of WDD disease. In the context of the increased exposure of crops to insect vectors (recent ban of neonicotinoids in Europe and climate change), these findings represent a first step in the understanding of the impact of co-infections of BYDV-PAV and WDV on the biological and ecological parameters of diseases caused by these viruses. Next steps would be the characterization of molecular mechanism(s) underlying BYDV-PAV – WDV interactions and the evaluation of the net effect of the sympatry between these viruses and their respective vectors on the epidemiology of YDD and WDD in cereal fields.

## Data availability statement

The raw data supporting the conclusions of this article will be made available by the authors, without undue reservation.

## Author contributions

TA, MS and EJ conceived and designed the experiment. TA, MS, LK, KG and EJ performed the experiment. TA analysed the data. TA and EJ wrote the article. All the authors read and approved the submitted version of the manuscript.

## Supplementary material

Supplementary Figure 1 Accumulation of BYDV-PAV **(A)** and WDV **(B)** in singly and co-infected plants. Singly and simultaneously inoculated wheat plants (7 days-old plants at inoculation; 5 viruliferous *R. padi* and/or 5 P*. alienus* for 24 h IAP) were sampled at 2, 5, 8,12 days after inoculation (DAI). Then, the viral load of BYDV-PAV and WDV was evaluated in each plant by (RT)-qPCR. For BYDV-PAV **(A)** and WDV **(B)**, viral load measured in singly and co-infected are presented irrespectively to the DAI. Box plots show outliers (dots), 10–90% percentiles (whiskers), 25–75% percentiles (boxes), median (lines) and mean (black point). These parameters were calculated using BYDV-PAV infected (*n*= 60), WDV infected (*n*= 83) and coinfected (*n*= 42) plants from three repetitions of the experimental design. Asterisks (*) were obtained after statistical analysis of the data (ANOVA). For a virus species, asterisk illustrates significant difference between singly and co-infected plants. *: *P*≤ 0.05.

Supplementary Figure 2 Transmission rate (*T_Ci_*) from co-infected source plants. Co-infected wheat plants (7-days old at inoculation; 5 viruliferous *R. padi* and 5 P*. alienus* for 24 h IAP) were used as *source* plants in transmission experiments carried out at 7 and 21 days of infection (DoI). Virus-free insects were deposited on infected *source* plants for an acquisition access period (BYDV-PAV: 6 hours; WDV: 24 hours), before being transferred on 7-days old *test* plants (1 *R. padi* or 2 P*. alienus* per *test* plant). For each co-infected *source* plant, the sanitary status of *test* plants was individually tested to calculate the *T_Ci_*. Histogram bars and error bars represent mean and standard errors of the *T_Ci_*, respectively. These two parameters were calculated using co-infected plants (BYDV-PAV; 7 DAI: *n*= 20, 21 DAI: *n*= 16; WDV; 7 DAI: *n*= 16, 21 DAI: *n*= 21) obtained after three repetitions of the experimental design. Letters (in bold for co-infected plants used to evaluate the *T_Ci_* of WDV) were obtained after statistical analysis of the data (GLM (family binomial) followed by EMM *post-hoc* test). For a virus species, different letters indicate a significant difference (α = 0.05) between DoI.

Supplementary Figure 3 Co-infection rates (*I_seq_*) on pre-infected wheat plants. Plants (7-days old at inoculation, 2 virus-free *R. padi* or *P. alienus* for 24 h IAP) were pre-infected by BYDV-PAV or WDV, before being used in a second inoculation step (2 viruliferous *P. alienus* or *R. padi* for 24 h IAP) at 2, 5, 8 and 12 days after pre-infection (Length of pre-infection period: LoP). Twenty-one days after the second inoculation, plants were individually tested by DAS-ELISA against BYDV-PAV and WDV to calculate *I_Seq_* of each treatment (W/B: in grey; B/W: in white). Experiments were carried out three times with series of 20 plants/virus species/treatment/LoP. Histogram bars and error bars represent mean and standard error of *I_Seq_* values, respectively. Letter (a) were obtained after statistical analysis of the data (GLM (family binomial) followed by EMM *post-hoc* test). For a treatment (in bold for B/W treatment), different letters indicate a significant difference (α = 0.05) between LoP.
