# Supplementary figures and images for: Asymmetric interactions between barley yellow dwarf virus -PAV and wheat dwarf virus in wheat
Source: Front Plant Sci. 2023 Jul 11;14:1194622. doi: 10.3389/fpls.2023.1194622 (PMC10366370; doi:10.3389/fpls.2023.1194622)

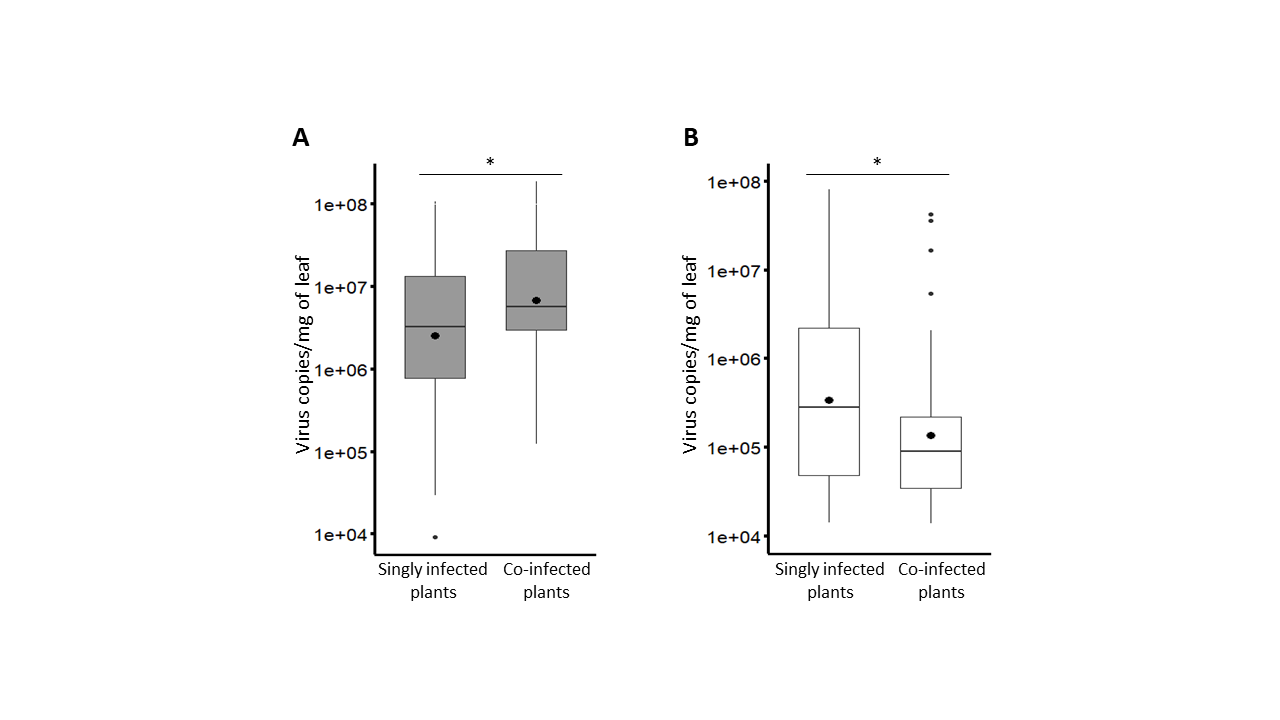

Supplement: Supplementary Figure 1 — Accumulation of BYDV-PAV (A) and WDV (B) in singly and co-infected plants. Singly and simultaneously inoculated wheat plants (7 days-old plants at inoculation; 5 viruliferous R. padi and/or 5 P. alienus for 24 h IAP) were sampled at 2, 5, 8,12 days after inoculation (DAI). Then, the viral load of BYDV-PAV and WDV was evaluated in each plant by (RT)-qPCR. For BYDV-PAV (A) and WDV (B), viral load measured in singly and co-infected are presented irrespectively to the DAI. Box plots show outliers (dots), 10–90% percentiles (whiskers), 25–75% percentiles (boxes), median (lines) and mean (black point). These parameters were calculated using BYDV-PAV infected (n= 60), WDV infected (n= 83) and coinfected (n= 42) plants from three repetitions of the experimental design. Asterisks (*) were obtained after statistical analysis of the data (ANOVA). For a virus species, asterisk illustrates significant difference between singly and co-infected plants. *: P≤ 0.05. [file Image_1.tif]

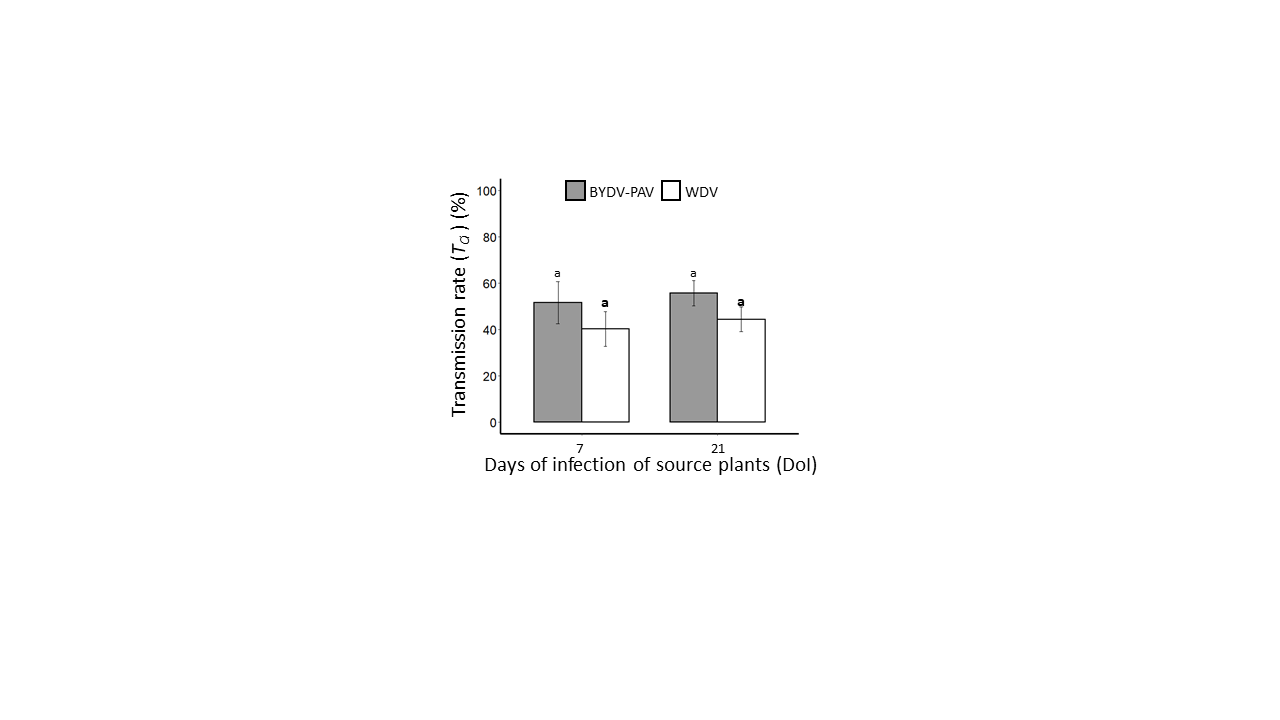

Supplement: Supplementary Figure 2 — Transmission rate (TCi ) from co-infected source plants. Co-infected wheat plants (7-days old at inoculation; 5 viruliferous R. padi and 5 P. alienus for 24 h IAP) were used as source plants in transmission experiments carried out at 7 and 21 days of infection (DoI). Virus-free insects were deposited on infected source plants for an acquisition access period (BYDV-PAV: 6 hours; WDV: 24 hours), before being transferred on 7-days old test plants (1 R. padi or 2 P. alienus per test plant). For each co-infected source plant, the sanitary status of test plants was individually tested to calculate the TCi . Histogram bars and error bars represent mean and standard errors of the TCi , respectively. These two parameters were calculated using co-infected plants (BYDV-PAV; 7 DAI: n= 20, 21 DAI: n= 16; WDV; 7 DAI: n= 16, 21 DAI: n= 21) obtained after three repetitions of the experimental design. Letters (in bold for co-infected plants used to evaluate the TCi of WDV) were obtained after statistical analysis of the data (GLM (family binomial) followed by EMM post-hoc test). For a virus species, different letters indicate a significant difference (α = 0.05) between DoI. [file Image_2.tif]

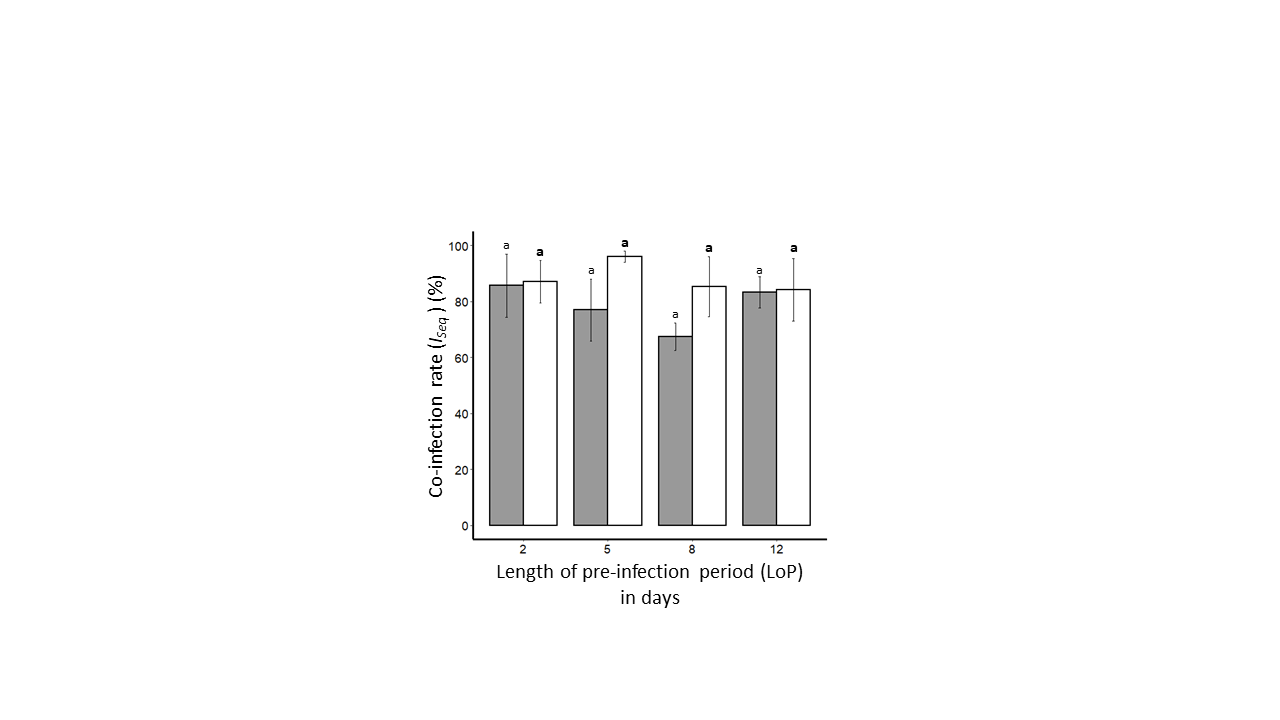

Supplement: Supplementary Figure 3 — Co-infection rates (Iseq ) on pre-infected wheat plants. Plants (7-days old at inoculation, 2 virus-free R. padi or P. alienus for 24 h IAP) were pre-infected by BYDV-PAV or WDV, before being used in a second inoculation step (2 viruliferous P. alienus or R. padi for 24 h IAP) at 2, 5, 8 and 12 days after pre-infection (Length of pre-infection period: LoP). Twenty-one days after the second inoculation, plants were individually tested by DAS-ELISA against BYDV-PAV and WDV to calculate ISeq of each treatment (W/B: in grey; B/W: in white). Experiments were carried out three times with series of 20 plants/virus species/treatment/LoP. Histogram bars and error bars represent mean and standard error of ISeq values, respectively. Letter (a) were obtained after statistical analysis of the data (GLM (family binomial) followed by EMM post-hoc test). For a treatment (in bold for B/W treatment), different letters indicate a significant difference (α = 0.05) between LoP. [file Image_3.tif]
