# Supplementary material for: Asymmetric interactions between barley yellow dwarf virus -PAV and wheat dwarf virus in wheat
Source: Front Plant Sci. 2023 Jul 11;14:1194622. doi: 10.3389/fpls.2023.1194622 (PMC10366370; doi:10.3389/fpls.2023.1194622)
Supplement: Supplementary file 4 [file Table_1.pdf]

Supplementary table 1. Construction of GLM and ANOVA analyses and numeric data. First part**.**

Test	Data	Factors	*Df*	*F*	*P**
Fig. 1 (WDV)		Plant age at inoculation	4	2.47	0.11
Fig. 1 (BYDV-PAV)		Plant age at inoculation	4	11.03	6.0×10^{-9}
Fig. 2 (WDV)		Days after inoculation (DAI)	3	9.44	2.11×10^{-5}
Fig. 2 (BYDV-PAV)		DAI	3	0.16	0.92
Fig. 3A (WDV)		Days of infection (DoI)	1	25.14	1.5×10^{-5}
Fig. 3A (BYDV-PAV)		DoI	1	0.46	0.50
Fig. 3B (WDV)		Days of infection (DoI)	1	24.95	1.4×10^{-5}
Fig. 3B (BYDV-PAV)		DoI	1	0.39	0.53
Fig. 4A (WDV)	GLM binomial	Plant age at inoculation	4	1.42	0.22
Fig. 4A (BYDV-PAV)	GLM binomial	Plant age at inoculation	4	0.39	0.81
Fig. 4C	GLM binomial	Plant age at inoculation	4	1.84	0.12
Table 3 and Supplementary Fig.1 (WDV)	ANOVA	Infection type	1	6.55	0.01
		DoI	3	12.17	5.5×10^{-7}
		Infection type : DoI	3	0.71	0.55
Table 3 and Supplementary Fig.1 (BYDV-PAV)	ANOVA	Infection type	1	5.74	0.02
		DoI	3	0.37	0.77
		Infection type : DoI	3	0.33	0.80
Supplementary Fig. 2 (WDV)	GLM binomial	DoI	1	0.39	0.52
Supplementary Fig. 2 (BYDV-PAV)	GLM binomial	DoI	1	0.17	0.67
Fig. 5 (7 DoI; WDV)	GLM binomial	Infection type	1	1.55	0.21
		Experimental repetition (ER)	2	10.55	9.0×10^{-5}
		Infection type : ER	2	0.71	0.49
Fig. 5 (21 DoI; WDV)	GLM binomial	Infection type	1	18.19	1.3×10^{-5}
		ER	2	1.61	0.13
		Infection type : ER	2	3.02	0.11
Fig. 5 (7 DoI; BYDV-PAV)	GLM binomial	Infection type	1	1.38	0.24
		ER	2	3.78	0.02
		Infection type : ER	2	2.13	0.12
Fig. 5 (21 DoI; BYDV-PAV)	GLM binomial	Infection type	1	0.01	0.91
		ER	2	2.69	0.07
		Infection type : ER	2	0.8	0.45
Table 4 (WDV)	ANOVA	Infection type	1	5.49	0.02
		DoI	1	36.00	9.7×10^{-8}
		Infection type : DoI	1	4.67	0.03
Table 4 (BYDV-PAV)	ANOVA	Infection type	1	0.21	0.68
		DoI	1	0.30	0.59
		Infection type : DoI	1	0.17	0.69
Table 5 (WDV)	GLM binomial	Mock inoculation	1	3.63	0.08
		Days after mock inoculation (DaMI)	1	8.44	0.008
		Mock inoculation : DaMI	1	1.07	0.74
Table 5 (BYDV-PAV)	GLM binomial	Mock inoculation	1	0.33	0.56
		DaMI	1	8.62	0.008
		Mock inoculation : DaMI	1	2.74	0.21
Supplementary Fig. 3 (WDV)	GLM binomial	Length of pre-infection (LoP)		1.89	0.16
Supplementary Fig. 3 (BYDV-PAV)	GLM binomial	LoP		1.37	0.24

*: Significance level was fixed at 0.05 for all statistical test.

Supplementary table 1. Construction of GLM and ANOVA analyses and numeric data.. Second part**.**

Test	Data	Factors	*Df*	*F*	*P*
Fig. 6 (2 LoP; WDV)	GLM binomial	Infection type	1	0.21	0.64
Fig. 6 (5 LoP; WDV)	GLM binomial	Infection type	1	12.30	$8.1 x10^{-5}$
Fig. 6 (8 LoP; WDV)	GLM binomial	Infection type	1	10.4	0.001
Fig. 6 (12 LoP; WDV)	GLM binomial	Infection type	1	7.59	0.006
Fig. 6 (2 LoP; BYDV-PAV)	GLM binomial	Infection type	1	0.3	0.62
Fig. 6 (5 LoP; BYDV-PAV)	GLM binomial	Infection type	1	0.66	0.51
Fig. 6 (8 LoP; BYDV-PAV)	GLM binomial	Infection type	1	5.92	0.01
Fig. 6 (12 LoP; BYDV-PAV)	GLM binomial	Infection type	1	7.52	0.005

*: Significance level was fixed at 0.05 for all statistical test.
